# Nanoarchitectonics Revolution and Evolution: From Small Science to Big Technology

**DOI:** 10.1002/smsc.202000032

**Published:** 2020-12-06

**Authors:** Katsuhiko Ariga

**Affiliations:** ^1^ World Premier International (WPI) Research Center for Materials Nanoarchitectonics (MANA) National Institute for Materials Science (NIMS) 1-1 Namiki Tsukuba 305-0044 Japan; ^2^ Department of Advanced Materials Science Graduate School of Frontier Sciences The University of Tokyo 5-1-5 Kashiwanoha Kashiwa Chiba 277-8561 Japan

**Keywords:** interfaces, living cells, molecular machines, nanoarchitectonics, nanotechnology, self-assembly

## Abstract

Along with the progresses of material syntheses, the importance of structural regulation is realized to rationally improve the efficiencies and specificities in target functions. Small science is necessary for advanced material systems. A novel concept, nanoarchitectonics, to combine nanotechnology with the other scientific disciplines to synthesize a functional material system with contributions of small objects, nano‐units, is recently proposed. Based on facts and knowledge in nanoscale objects explored by nanotechnology, functional material systems are constructed using nano‐units with the aid of the other research fields, such as organic chemistry, supramolecular chemistry, materials science, and biology. The introduction of nanoarchitectonics essences to material construction can produce unusual functional systems, such as brain‐like information processing based on atomic‐level reactions, diffusions, and aggregations. Probe‐tip‐mediated organic reactions are also possible with precise site selectivity. The coupling of equilibrium self‐assemblies and non‐equilibrium fabrication processes results in variously structured and hierarchical functional structures even from simple 0D nano‐units such as fullerenes. Especially, interfacial nanoarchitectonics directly bridge nanoscopic functions and macroscopic actions, including facile contact with nanostructures and living cells. This review article overviews nanoarchitectonics from origin to future, from atoms to materials, and from small science to big technology.

## Graphical abstract

This review explains the basics of nanoarchitectonics based on historical backgrounds and comparisons with well‐known existing concepts, such as nanotechnology and self‐assembly. Revolution from nanotechnology to nanoarchitectonics enables us to develop methodology for material production comparable to an evolution process in nature. The establishment of processes equivalent to billion‐years of natural evolution within a few decades is the final goal of nanoarchitectonics.

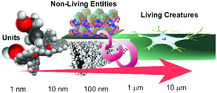

## 1 Introduction

Contributions of science for small structures are not small in current technology. Even for large‐size objects and huge quantity materials, their functions are often regulated through precise controls of their internal small structures and components. Small science has big effects.

Upon various social demands, scientists have to produce functional materials and systems, especially for energy,^[^ ^1^ ^]^ environmental,^[^ ^2^ ^]^ and biomedical^[^ ^3^ ^]^ problems. Fundamental strategies on developments of functional materials systems simply reply on synthetic approaches related to organic synthesis,^[^ ^4^ ^]^ polymer chemistry,^[^ ^5^ ^]^ and materials processes.^[^ ^6^ ^]^ Along with the progresses of material syntheses, the importance of structural regulation is realized to rationally improve the efficiencies and specificities in target functions.^[^ ^7^ ^]^ Small science is necessary for advanced material systems.

Great successful examples of highly advanced material systems with essences of small science can be seen in many biological systems.^[^ ^8^ ^]^ Precise arrangement of amino acid residues within enzymatic pockets resulted in reactions (often sequential reactions) with high efficiency and specificity under mild ambient conditions. Programmed sequences in DNA strands are transcripted into protein structures that play indispensable roles. Rational dispositions of chromophores and proteins at cell membranes and within cells lead to sophisticated functions, such as photosynthesis and signal transductions. Although small units have simple structures, their organizations bring energy/electron transfers and sequential interaction/reaction with vectorial and direction‐specified natures as well as high efficiencies and specificities. Their performances including conversion efficiency often exceed those for man‐made systems. Biological systems with essences of small science sometimes become ideal models for functional material systems. Several biomimetic and bioinspired approaches for functional material systems, including DNA‐based technologies,^[^ ^9^ ^]^ nanomaterials constructions,^[^ ^10^ ^]^ and molecular assemblies,^[^ ^11^ ^]^ have been actually investigated.

As seen in the examples mentioned earlier, rational production of functional materials needs contribution from small science. Necessary items for these processes can be simply summarized as follows: 1) synthetic approaches to fabricate atomic arrangement, molecules, and nanomaterials as unit components; 2) techniques for observation, manipulation, and evaluation of nanostructured components; 3) methodologies for construction of these nano‐units into functional materials; and 4) efforts on further developments for application of the fabricated materials.

These processes are, of course, included in the existing sciences and technologies. Huge accomplishments on organic synthesis, polymer chemistry, and materials sciences can be subjected to item 1).^[^ ^12^ ^]^ Various sciences on self‐assembly/self‐organization are working for item 3)^[^ ^13^ ^]^ as well as physical nano/micro‐fabrications, such as various lithographic techniques^[^ ^14^ ^]^ and interfacial supramolecular processes, including the self‐assembled monolayer (SAM) method,^[^ ^15^ ^]^ Langmuir–Blodgett (LB) techniques,^[^ ^16^ ^]^ and layer‐by‐layer (LbL) assembly.^[^ ^17^ ^]^ Based on these research accomplishments, various efforts for item 4) have been made in various fields, including energy,^[^ ^18^ ^]^ environmental,^[^ ^19^ ^]^ and biomedical applications.^[^ ^20^ ^]^

Important essences of small science (item 2)) were added by rapid prosperities of nanotechnology.^[^ ^21^ ^]^ Although the other items have rather chemistry‐oriented natures, item 2) with nanotechnology introduces physics‐based thinking to these preceding chemistry‐based approaches. As shown later in this article, nanotechnological approaches may even add new pages on organic synthesis, which is believed as purely chemistry‐dominated field. The appearance of nanotechnology gradually changes basic thought on production of functional materials. Therefore, it is high time to establish a novel concept to combine nanotechnology with the other scientific disciplines to synthesize the functional material system with contributions of small science, nanotechnology.

**Figure 1:**
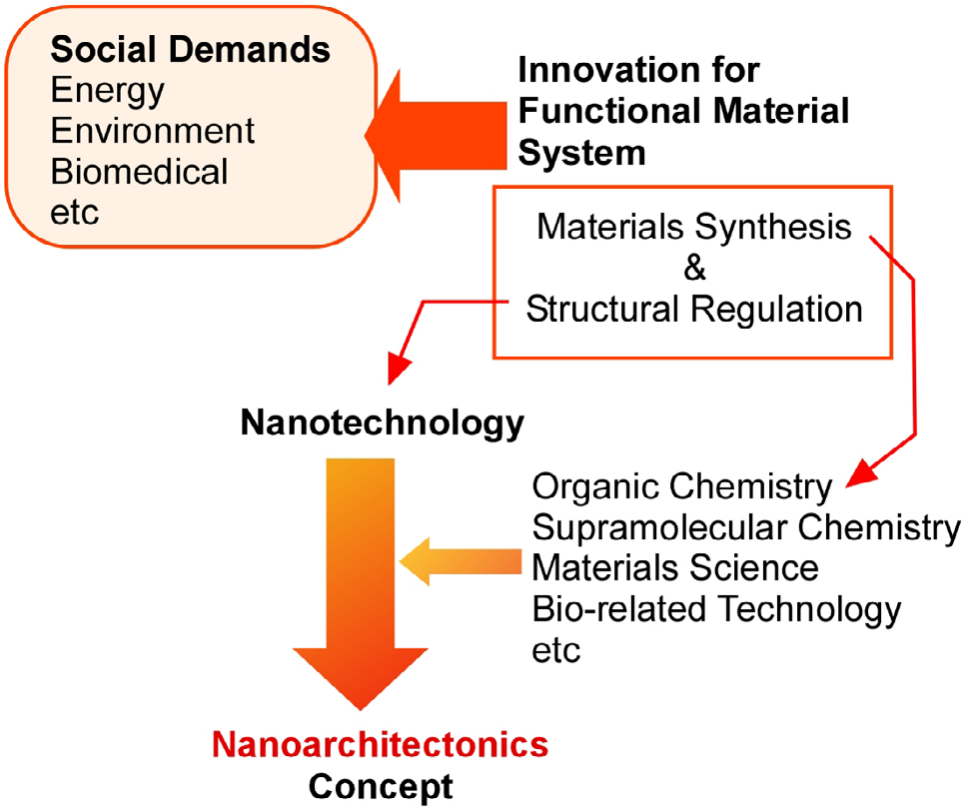
The nanoarchitectonics approaches to produce the functional material system upon structural construction from nano‐units through combining nanotechnology concept and the other research fields, such as organic chemistry, supramolecular chemistry, materials science, and bio‐related technology.

This task is taken by a novel concept, nanoarchitectonics,^[^ ^22^ ^]^ that was initially proposed by Aono and co‐workers^[^ ^23^ ^]^ such as the initiation of nanotechnology by Feynman.^[^ ^24^ ^]^ The nanoarchitectonics approaches are supposed to produce the functional material system upon structural construction from nano‐units through combining the nanotechnology concept and the other research fields, such as organic chemistry, supramolecular chemistry, materials science, and bio‐related technology (**Figure** **1**).^[^ ^25^ ^]^ Although working fields of nanotechnology are mainly focused on evaluation and manipulation of nanoscale objects, the nanoarchitectonics concept intends to connect nanoscale science to materials technology from small science to big technology.^[^ ^26^ ^]^

Because the nanoarchitectonics concept is general and applicable to various research fields, purpose‐oriented research papers with the nanoarchitectonics concept have been recently published in fields of materials synthesis,^[^ ^27^ ^]^ structure organization,^[^ ^28^ ^]^ catalyst,^[^ ^29^ ^]^ sensors,^[^ ^30^ ^]^ energy,^[^ ^31^ ^]^ environment,^[^ ^32^ ^]^ devices,^[^ ^33^ ^]^ basic biochemistry,^[^ ^34^ ^]^ and biomedical applications.^[^ ^35^ ^]^ Unlike these previous publications, this article explains the fundamental aspects of nanoarchitectonics based on historical backgrounds and comparisons with well‐known existing concepts, such as nanotechnology and self‐assembly. These descriptions are mainly formatted into two flows of 1) revolution from nanotechnology to nanoarchitectonics and 2) evolution from atom/molecule to material with nanoarchitectonics. Some hot topics with nanoarchitectonics essences are occasionally included. Finally, expectation of near future progresses of the nanoarchitectonics research and final goals of the nanoarchitectonics research are discussed in perspective section.

## 2 Revolution from Nanotechnology to Nanoarchitectonics

**Figure 2:**
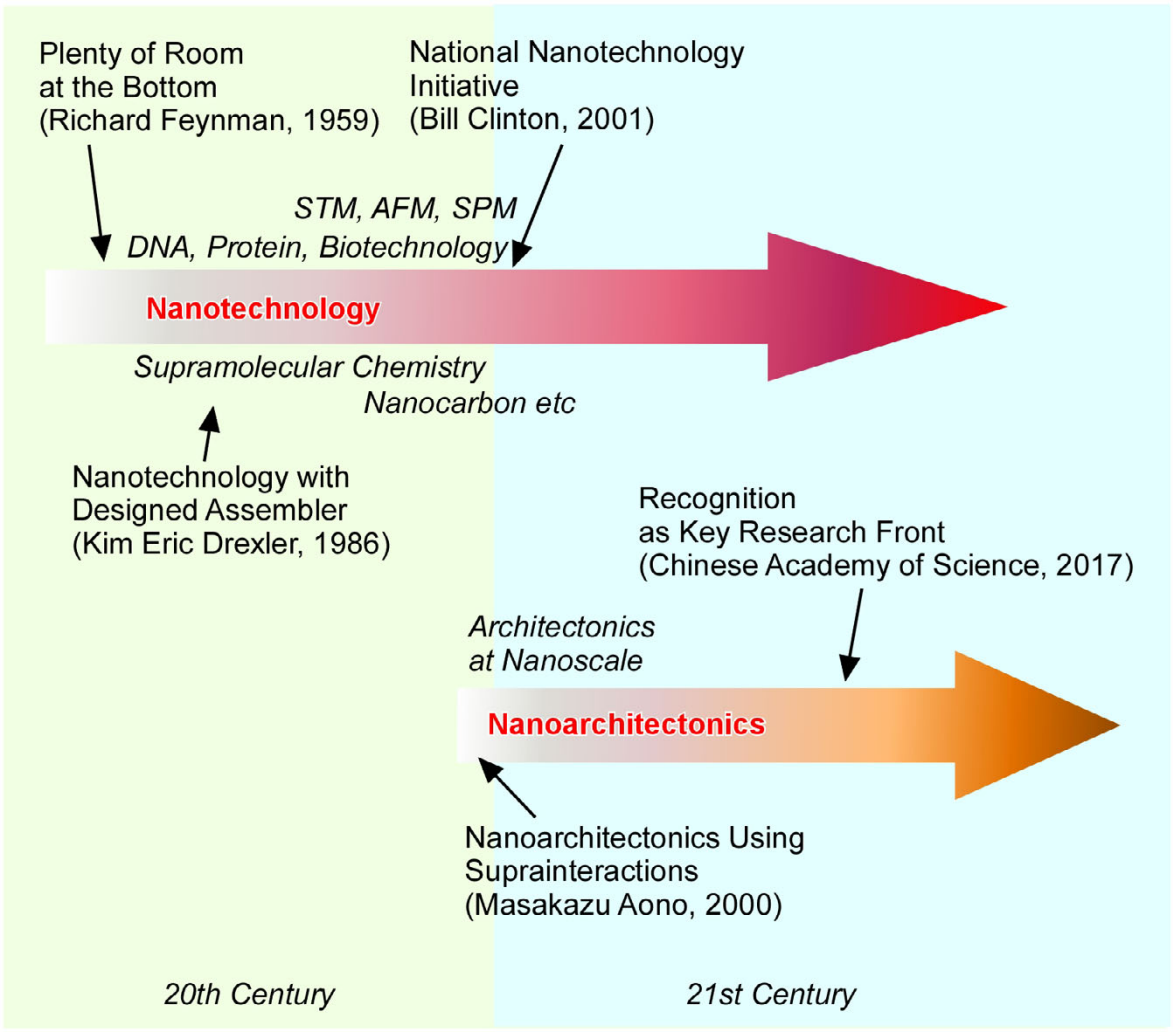
Historical background on paradigm shifts from nanotechnology to nanoarchitectonics.

First of all, historical facts on paradigm shifts from nanotechnology to nanoarchitectonics are briefly summarized here, although such backgrounds are often fragmentally mentioned (**Figure** **2**). The concept of nanotechnology is said to be initiated by Feynman in his lecture entitled There's Plenty of Room at the Bottom in 1959.^[^ ^36^ ^]^ Miniaturizations including atom‐level manipulations and their new sciences were expected. Inventions of scanning tunneling microscopy (STM) and related scanning probe microscopies (SPM) in atomic force microscopy (AFM) enable us to observe and manipulate atom‐level and molecular‐level objects.^[^ ^37^ ^]^ In fact, manipulation of artificial arrangements of Xe atoms to draw “IBM” was demonstrated. In parallel, various techniques for micro‐ and nano‐fabrications were developed. Accordingly, nanotechnology has been paid much attention as the 21st century technology revolutions that was also symbolized with declaration by US president Bill Clinton in 2001 to promote nanotechnology, the National Nanotechnology Initiative.

Another conceptual proposal was made by Eric Drexler in 1986.^[^ ^38^ ^]^ In his book entitled Engines of Creation: The Coming Era of Nanotechnology, molecular and biomolecular nanotechnology with designed assembler to produce functional systems were described. Not limited to mechanical assembly concept, scientific developments in biology and chemistry, such as promoted understanding and technology developments of biological central dogma from DNAs to proteins,^[^ ^39^ ^]^ conceptual initiation and demonstration of molecular machines,^[^ ^40^ ^]^ and exploration of self‐assembly and self‐organization in supramolecular chemistry,^[^ ^41^ ^]^ may possibly support this idea as core methodologies. These trends were recognized as bottom‐up‐type nanotechnology, especially in the fields of chemistry and biochemistry.

These concepts and strategies can be more generalized as construction of functional material systems from nano‐level building units. A term of architectonics related to small objects was used in an article entitled Architectonic Quantum Dot Solids by Heath and co‐workers in 1999, University of California Los Angeles (UCLA).^[^ ^42^ ^]^ The use of novel conceptual term, nanoarchitectonics, was initiated in 2000 by Aono^[^ ^43^ ^]^ who organized international symposium, the first International Symposium on *Nanoarchitectonics* Using Suprainteractions in Tsukuba, Japan. This is the first initiation of the new concept, nanoarchitectonics.^[^ ^44^ ^]^ Aono intends to combine nanotechnology initiated by Feynman with the other scientific disciplines to establish total methodology to create functional materials with nano‐scale units. The first appearance of the term of nanoarchitectonics in title of papers in scientific journals was made by Hecht of the Freie Universität Berlin, Germany, in his paper titled, *Welding, Organizing, and Planting Organic Molecules on Substrate Surfaces–Promising Approaches towards Nanoarchitectonics from the Bottom Up*.^[^ ^45^ ^]^ At the same time, several research organization and projects were initiated with the term on nanoarchitectonics. A research center, Interfacial Nanoarchitectonics, led by Toshimi Shimizu was initiated in 2003, at the National Institute of Advanced Industrial Science and Technology in Tsukuba. A research center, Functional Engineered Nano Architectonics, was opened in 2003 at UCLA. The founder of nanoarchitectonics, Masakazu Aono, launched the World Premier International Research Center for Materials Nanoarchitectonics (WPI‐MANA) at the National Institute for Materials Science in Tsukuba in 2007. Along with starting of the 21st century, the nanoarchitectonics concept was emerged and has been developing. In 2017, nanoarchitectonics was recognized as one of the key research fronts in a report by Institutes of Science and Development and The National Science Library, Chinese Academy of Sciences with the aid of Clarivate Analytics.

Including all these backgrounds, the nanoarchitectonics covers huge size regions from atomic‐size nanoscience to practical‐level materials technology through materials productions from nano‐units. According to basic strategies indicated by Ariga and Aono, the following issues are considered in the nanoarchitectonics processes.^[^ ^46^ ^]^ 1) Reliable nanomaterials or nanosystems are created through organization of nanoscale structures, so‐called nano‐parts, even with some unavoidable uncertainties. 2) In these processes, the main players are not always the individual nano‐units, but their interactions play key roles to create novel functions. 3) Unexpected functionalities can be emerged as the results of assembly and organization of numerous nano‐units. 4) The new theoretical fields can supports these processes.

The nanoarchitectonics concept is supposed to proceed production of new functional material systems from the nano‐units through combination and selection of various processes, such as atom/molecular manipulation, organic molecular modification, self‐assembly and self‐organization, field‐assisted system rearrangement, microfabrication, materials processing, and bio‐related treatments.

Accumulation of the selected processes works on architecting of functional materials from nano‐parts. However, simple summation would not be exact expression of effect combination in nanoarchitectonics processes. Unlike microscopic and macroscopic regions, certain uncertainties, such as thermal fluctuations, statistical distributions, and quantum effects, are not always avoided in atomic/molecular/materials interactions at nanoscale size regions. Numerous nano‐units interact each other with some uncertainties to architect materials structures. Therefore, effects are harmonized (rather than summated) in nanoarchitectonics processes.^[^ ^47^ ^]^

**Figure 3:**
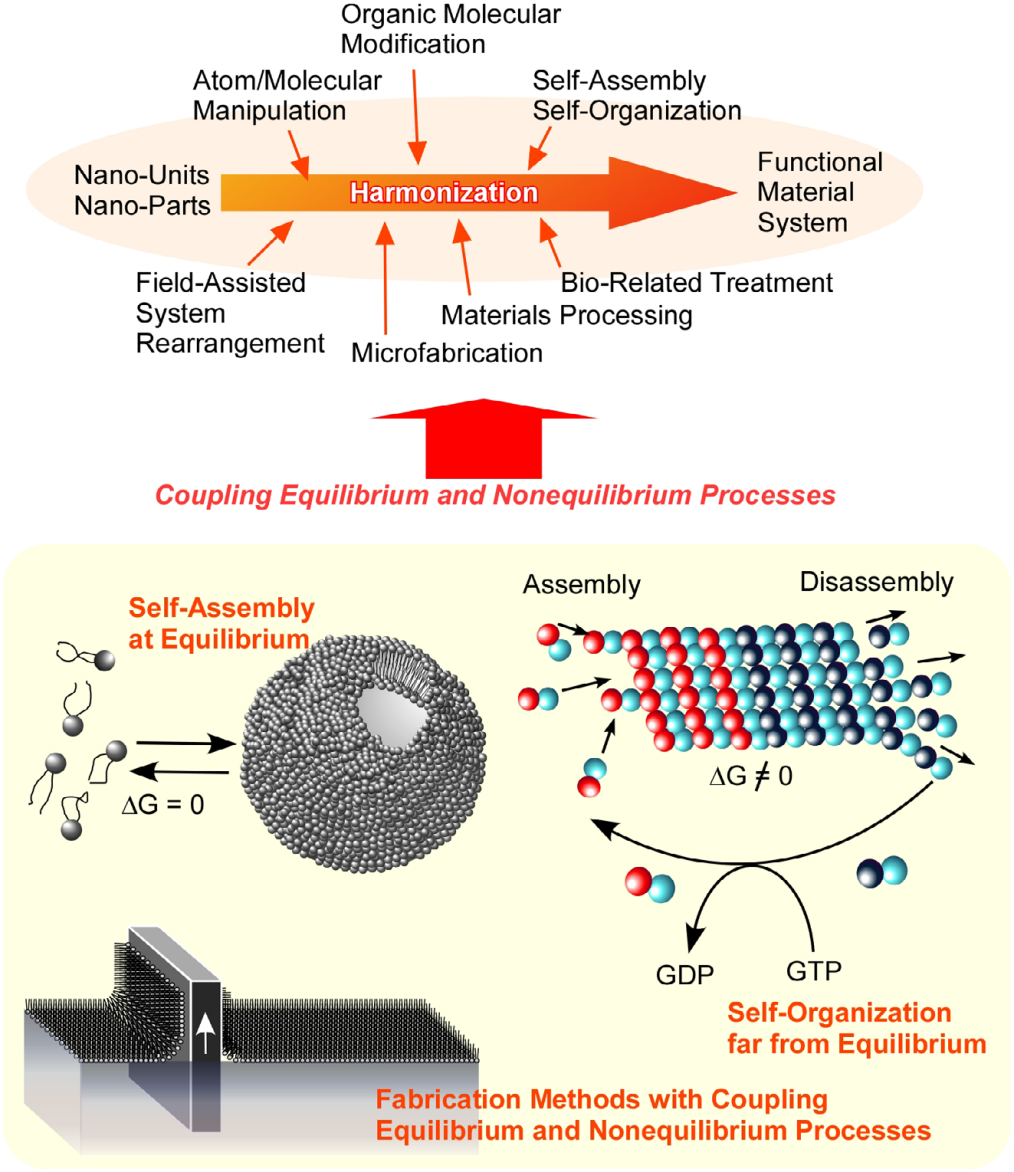
Nanoarchitectonics procedures including various energy consuming processes in step‐wise orders and/or harmonizing fashions as well as self‐assembly at equilibrium.

Self‐assembly and self‐organization play indispensable roles in nanoarchitectonics. Although self‐assembly is also central concept in organic‐based supramolecular chemistry, this concept can be widely applied to assembling processes of various nano‐units, including quantum objects, inorganic nanomaterials, and biological materials. Basic idea of self‐assembly processes is based on equilibrium without inputs of external energy and stimuli. Therefore, self‐assembly processes tend to provide symmetrical and simple‐structured assembly. In contrast, the self‐organization concept is used for structure architecting with energy flows from outsides of the system, and the self‐organization processes occur far from equilibrium. This nature of self‐organization is advantageous to architect asymmetric, vectorial, and hierarchical structures, which is universally used in biological structural organization. The nanoarchitectonics has strong feature similar to self‐organization. Nanoarchitectonics procedures can include various energy consuming processes in step‐wise orders and/or harmonizing fashions (**Figure** **3**).^[^ ^48^ ^]^ Integration of some artificial energy‐consuming processes and various fabrication processes into spontaneously occurring self‐assembly of nano‐units results in asymmetric and hierarchical architectures in the nanoarchitectonics process. Nanoarchitectonics approaches have nature rather similar to biological structure‐forming processes than conventional self‐assembly.^[^ ^49^ ^]^

## 3 Evolution from Atom/Molecule to Material with Nanoarchitectonics

**Figure 4:**
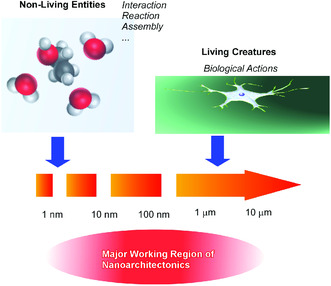
Major working size region for nanoarchitectonics similar to conversion point from non‐living entities to living creatures.

In this section, nanoarchitectonics approaches are overviewed according to size. If we consider all the materials including non‐living matters and living creatures from atomic level to visible level, boarder between non‐living matters and living creatures can be probably set at submicrometer to micrometer size (10^−7^ to 10^−6^ m). At one‐ or two‐order smaller size region (10^−9^ to 10^−8^ m), bio‐functional mechanisms with biomolecules are working under unavoidable influences of thermal fluctuations. Accumulation and harmonization of non‐living mechanisms are converted into living activities upon size increase from nanometer level to (sub)micrometer level. These working size regions are almost identical with those for production of functional materials systems from nano‐units in nanoarchitectonics approaches.^[^ ^50^ ^]^ It can be said that working principles of evolution from non‐living matters to living creatures are similar to those for nanoarchitectonics from nano‐units to functional materials (**Figure** **4**). The most distinct difference between biological evolution and nanoarchitectonics is probably their process time frames.^[^ ^51^ ^]^ Evolution of living creatures from simple molecules takes billions of years. On the other hand, the nanoarchitectonics concept was initiated only at the beginning of this century. Nanoarchitectonics aims to make materials evolutions similarly to biological evolution only within a few decades. In the following sections, some nanoarchitectonics‐featured research efforts are exemplified from atom level to materials size.

### 3.1 Atomic and Molecular‐Scale Nanoarchitectonics

The nanoarchitectonics concept can cover all the length scales from nano to visible range. As the bottom‐level nanoarchitectonics processes, examples on atomic and molecular‐scale nanoarchitectonics are shortly described in this section.

Aono and co‐workers have demonstrated in a series of their research on atomic switches that regulation of atom‐level arrangement is possibly connected to highly advanced function, brain‐like information conversion.^[^ ^52^ ^]^ In atomic switch devices, atom‐level motions, such as electrochemical reaction, atom rearrangement, and atom diffusion, can be converted to device‐level outputs of switching on and off of an electrical signals, which can be linked into neuromorphic computing structures.

**Figure 5:**
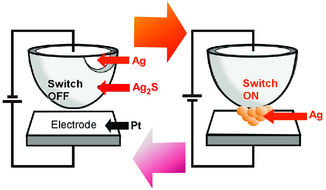
Basic design of typical atomic switch with Ag_2_S electrode and a Pt electrode separated with a gap of about 1 nm where the formation of ten atom‐level silver cluster through diffusion and rearrangement of silver atoms leads to electrical bridging between two electrodes to make the device switching on.

In basic design of typical atomic switch (**Figure** **5**), Ag_2_S is used as one electrode, and a Pt electrode is placed with a gap of about 1 nm where resistance is extremely high and no current flows between the electrodes.^[^ ^53^ ^]^ When a negative voltage is applied to the Pt electrodes, a tunnel current runs between the electrodes, resulting in the reduction of Ag^+^ inside the Ag_2_S electrode into Ag atom. The formation of ten atom‐level silver cluster through diffusion and rearrangement of silver atoms leads to electrical bridging between two electrodes to make the device switching on. Dissolution of the formed silver cluster upon oxidation of Ag to Ag^+^ into the Ag_2_S, results in switching off the same atomic switch.

**Figure 6:**
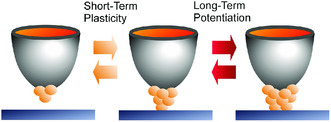
Inorganic synaptic devices with short‐term plasticity and long‐term potentiation capabilities upon electrochemical reactions and atom diffusion/aggregation in atomic switch.

These unit functions based on atomic‐level nanoarchitectonics upon electrochemical reactions and atom diffusion/aggregation lead to inorganic synaptic devices with short‐term plasticity and long‐term potentiation capabilities (**Figure** **6**).^[^ ^54^ ^]^ Rearrangement of Ag atoms forms conductive linkages between the electrodes. For establishment of stable conductive bridge, multiple pulses are required while cross linkages are not formed in a single pulse. This feature was used to realize a synaptic device operation dependent on the frequency of the signal input. Application of input pulses to low frequency such as repetition with a 20 s interval results in the formation and dissolution of conductive Ag aggregate bridges. This dynamic regulation corresponds to short‐term plasticity. Upon the application of a short interval pulse (2 s), stable and long‐lasting conductive Ag bridges are formed. With the latter operations, long‐term potentiation is achieved. These performances are similar to human brain functions. Imputing repeated information is said to be important to fix memory. Repeated learning is crucial to fix the corresponding information in our brains. Further networking of numerous numbers of atomic switches in real material network of nanowires can lead to development on neuromorphic computing.^[^ ^55^ ^]^ Diffusion and aggregation of atoms as purely physicochemical phenomena can be converted to brain‐like functions.

Bottom‐level nanoarchitectonics examples are not limited to atomic nanoarchitectonics. Recent research on molecular nanoarchitectonics would change common sense of organic synthesis.^[^ ^56^ ^]^ Molecular conversion by organic reaction is an important key process in nanoarchitectonics methodology. However, traditional approaches in molecular conversions rely on organic synthesis mostly in solvents and sometimes at solid interfaces. These environments do not provide nano‐specific features, such as nano‐level spatiotemporal regulation. Unlike these common senses, novel approaches for organic reaction upon introduction of nanotechnology to organic conversion have been recently demonstrated using the advanced probe microscopic (SPM) techniques.^[^ ^57^ ^]^ With the aid of SPM, molecular modification in point‐by‐point fashion on molecules and materials becomes possible. While SPM have great contributions in imaging technology, the removal of specific atoms from a molecule and connection of another molecular unit can be done with actions of a probe of SPM. These process can be starting points to create nano‐units and their arranged parts for further nanoarchitectonics processes.

**Figure 7:**
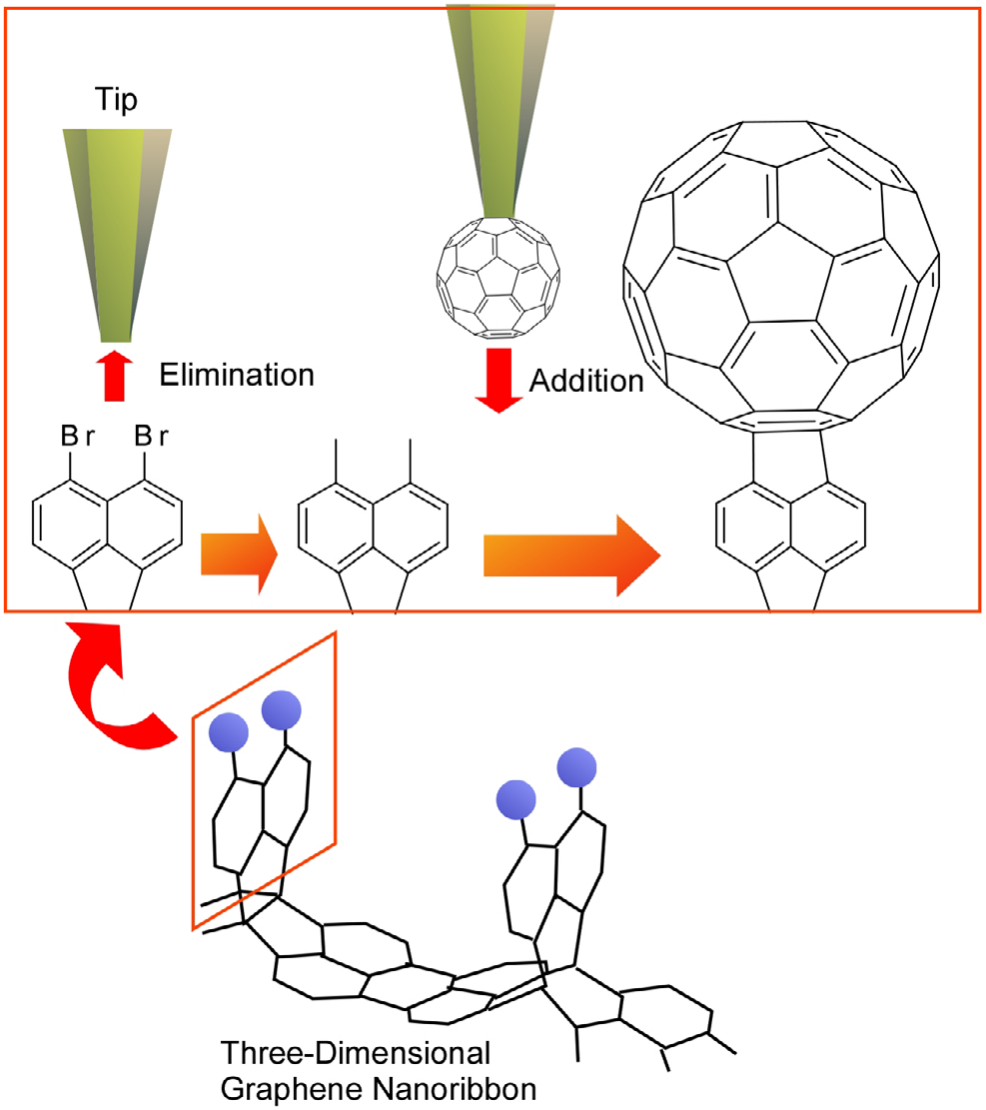
Site‐specific substitution of Br atoms by a fullerene molecule with an SPM tip at a graphene 3D nanotape on a Au(111) surface.

One significant example was recently demonstrated by Kawai and co‐workers who realized site‐specific substitution of Br atoms by a fullerene molecule with an SPM tip at a graphene 3D nanotape on a Au(111) surface (**Figure** **7**).^[^ ^58^ ^]^ Initially, Br atoms were removed from specific sites on the graphene nanotapes through moving the SPM tip to the vertical position (with interval of several hundred picometers) to the target Br atoms and applying a voltage of ≈2.5 V. Then, highly active unpaired electrons on carbon were resulted by the consequent Br atom removal. The activated unstable carbon sites were kept unreacted with the other substances at the surface at extremely low temperate under ultrahigh vacuum together with sterically advantageous 3D structures of the graphene nanotapes. A fullerene molecule was then attached to the active site with unpaired electrons to complete the substitution reaction. The fullerene molecules were previously adsorbed separately on the Au(111) surface, and a single C_60_ molecule was brought from C_60_ island to the activated site by an SPM tip. Covalent attachment of the C_60_ fullerene molecule to the desirable positions of the graphene nanotape was accomplished simply by pressing the C_60_ molecule to the activated diradical site. Organic reaction can be done in point‐by‐point fashion with SPM tip operations. The reactions do not always have to obey the rules of organic reactions and statistical probabilities.

**Figure 8:**
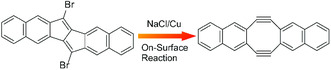
Synthesis of Sondheimer–Wong diyne from 6,13‐dibromopentaleno[1,2‐b:4,5‐b′]dinaphthalene using a probe tip on a Cu(111) surface covered with NaCl ultra‐thin film at 4.3 K.

Chemical reaction with localized probe tip allows synthesis and analysis of short‐term unstable products. Recently, Kawai and co‐workers demonstrated successful example on local probe chemistry for synthesis of Sondheimer–Wong diyne from 6,13‐dibromopentaleno[1,2‐b:4,5‐b′]dinaphthalene using a probe tip on a Cu(111) surface covered with NaCl ultra‐thin film at 4.3 K (**Figure** **8**).^[^ ^59^ ^]^ In the target reaction, the precursors, intermediates, and end products are structurally identified directly by differential conductance imaging using a CO‐functionalized chip. A plausible mechanism has been suggested that the reaction is accelerated by negatively charging the molecules for a short time by the tunnel current. Isolation of this molecular state with the insulated layer from metal is effective to preserve the negative charge for long period enough for molecular transition. Direction of the reaction regulated by local probe is opposite to the solution chemistry, but the probe‐mediated reaction has flexibility on local regulation of radical states.

As shown by the above‐mentioned examples, coupling of nanotechnology to organic chemistry would change common‐sense images of organic synthesis. Nanotechnology‐driven organic synthesis for molecular construction can be regarded as an organic nanoarchitectonics as a new trend in nanoarchitectonics strategies.

### 3.2 Nanoarchitectonics to Hierarchical Materials

As described earlier, the inclusion of nanotechnology essences to common phenomena in physics and chemistry, such as diffusion, aggregation, and reaction, opens new ways and thinking, although these matters are well matured. Similarly, rich possibilities and abundant challenges should still remain in phenomena and processes in length scales from atom/molecule to material when we combine nanotechnology essences to the traditional research fields, including supramolecular chemistry, materials science, and biology as nanoarchitectonics approaches.

**Figure 9:**
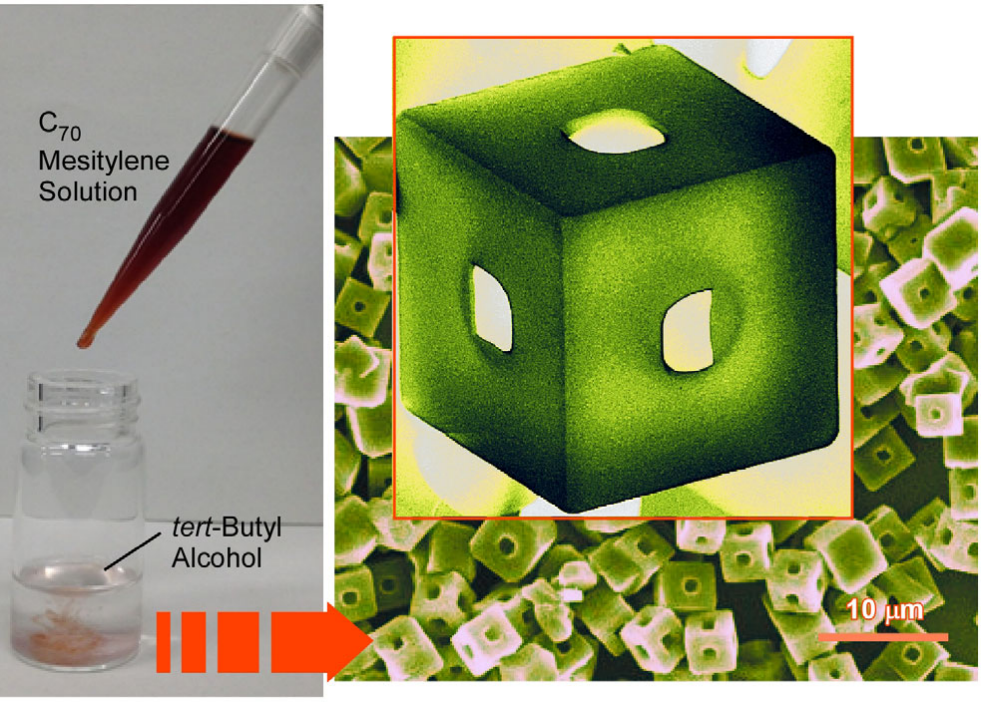
Precipitation of 0D single‐element molecular units, fullerenes (C_70_), at liquid–liquid interface to give integral structures, such as hole on cubes.

**Figure 10:**
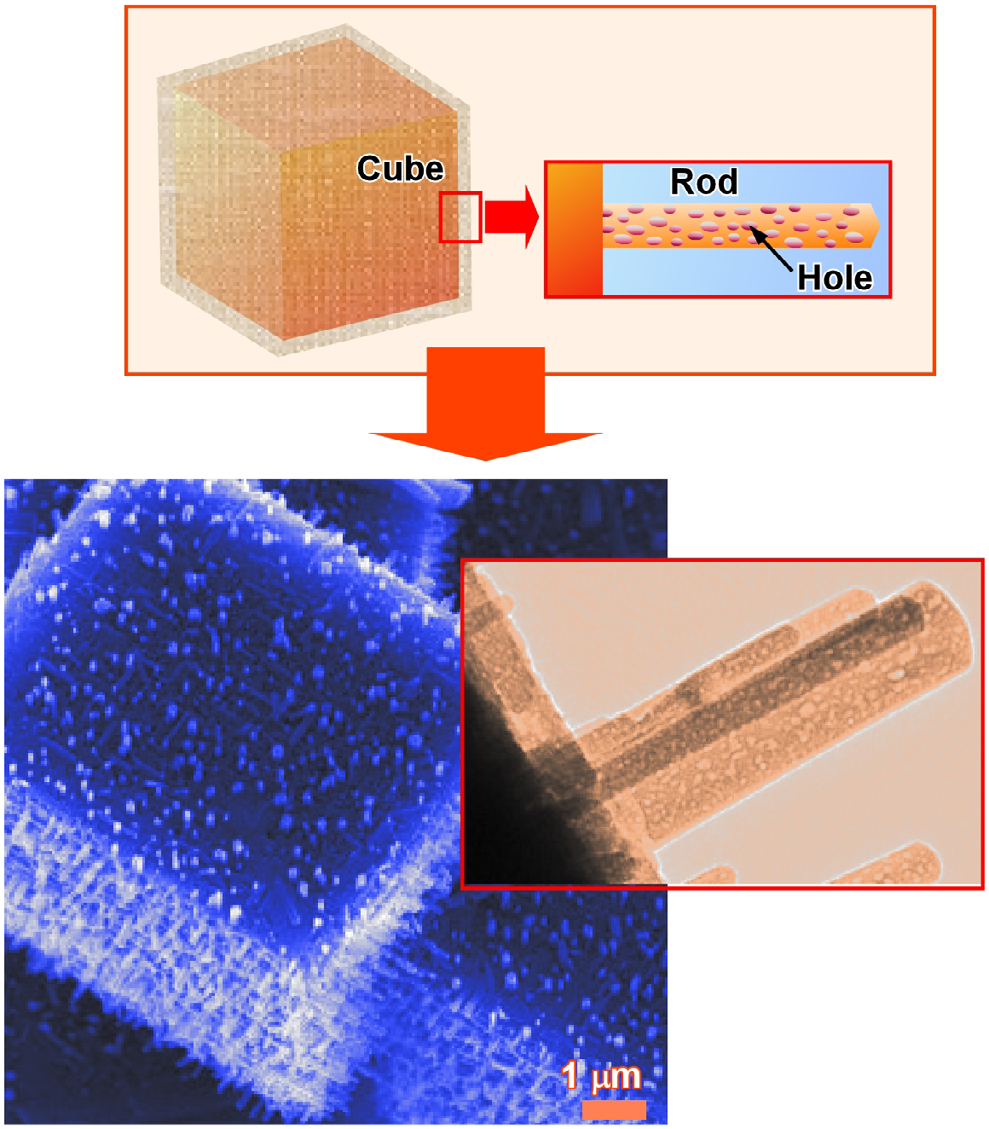
Hierarchic pore‐rod‐cube structures nanoarchitected through crystallization of C_70_ molecules at the interface between *tert*‐butyl alcohol and mesitylene, and the collected cubes followed by exposure to isopropanol at 25 °C.

Indeed, the simple self‐assembling and self‐organization processes have high potentials to form huge variety of structures. For example, precipitation of 0D single‐element molecular units, fullerenes (C_60_, C_70_, etc), at liquid–liquid interface gives various nano‐ and micro‐architectures, including 1D whiskers, rods, and tubes,^[^ ^60^ ^]^ 2D nanosheets with various shapes,^[^ ^61^ ^]^ 3D cubes,^[^ ^62^ ^]^ other interdimensional structures,^[^ ^63^ ^]^ and integral structures, such as hole on cubes (**Figure** **9**).^[^ ^64^ ^]^ The formation of these nanoarchitectures can be done by conventional lab‐bench procedures through simple physical principles of molecular dissolution, precipitation, aggregation, and crystallization. The addition of processes with external factors, including washing‐out particular components, solvent‐contacting phase transformation, sonication, and solvent evaporation, induces shifts from equilibrium to produce complicated, integrated, and hierarchic structures. For example, hierarchic pore‐rod‐cube structures can be nanoarchitected (**Figure** **10**).^[^ ^65^ ^]^ The 3D cubic objects were obtained through crystallization of C_70_ molecules at the interface between *tert*‐butyl alcohol and mesitylene, and the collected cubes were then exposed to isopropanol at 25 ^°^C. The latter process resulted in the growth of C_70_ rods perpendicularly to the cube surface through conversion of assembled structures. Because each nanorod has nanoporous nature, the hierarchical structure with nanopore, nanorods, and microcube (in order from smaller dimension to larger dimension) can be completed with these nanoarchitectonics procedures with equilibrium interfacial aggregation and non‐equilibrium solvent washing. The architected hierarchical structures exhibited promoted electrochemical performances and advanced sensing capability to aromatic guest gasses because of enhanced active surface area.

**Figure 11:**
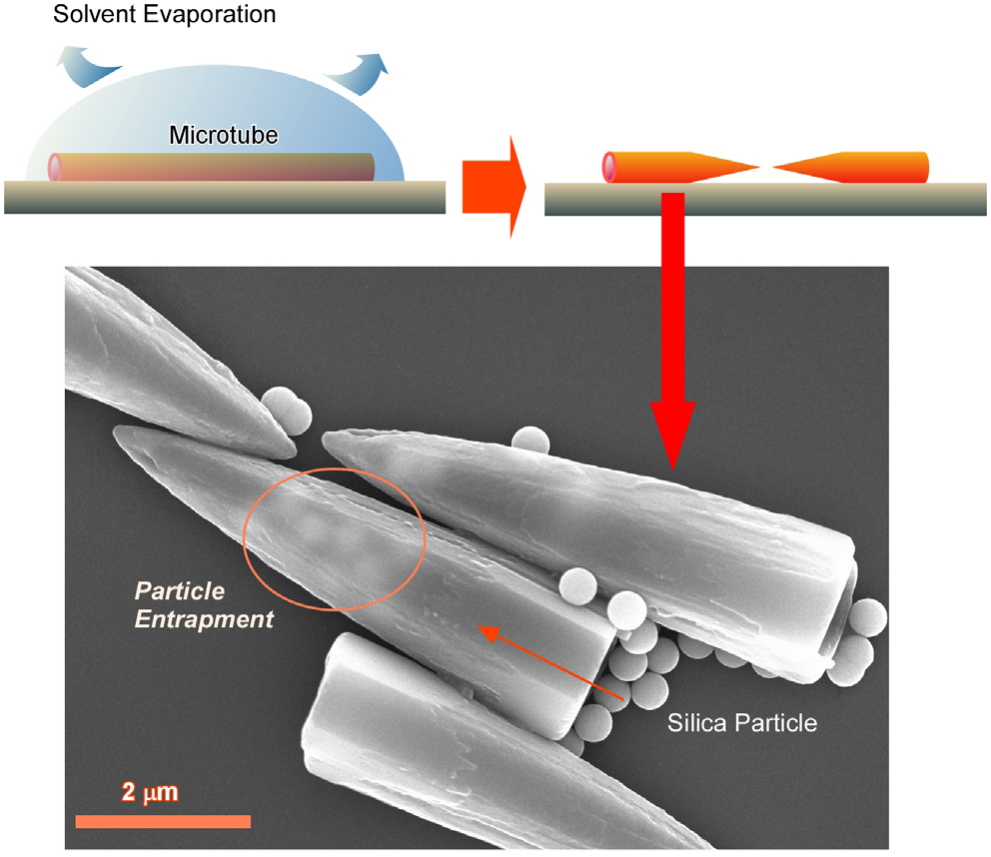
Transformation from fullerene (C_60_ and C_70_ mixture) microtubs to fullerene microhorns upon solvent evaporation and their particle trap capability.

Structures formed through noncovalent supramolecular assembly are capable of structural transformation upon additional process with external factor inputs. For example, transformation from fullerene (C_60_ and C_70_ mixture) microtubs to fullerene microhorns upon solvent evaporation was demonstrated (**Figure** **11**).^[^ ^66^ ^]^ Fullerene microtubes were initially prepared through aggregation of C_60_ and C_70_ molecules through the addition of *tert*‐butyl alcohol to C_60_/C_70_ mixed mesitylene solution. The obtained fullerene microtubes were then transformed into microhorns through the addition of *tert*‐butyl alcohol/mesitylene and gradual evaporation of the mixed solvents. One microtube produced two microhorns. The obtained microhorns possess structural features of half‐length of mother microtubes, hollow tubular end, and a sharp solid tip. This spontaneous structure transformation from microtubes to microhorns is originated in uneven distributions of C_70_ molecules in the mother microtube structures. C_70_ molecules with higher solubility to the mixed solvent preferentially distribute at the middle of the microtubes. Exposure of the microtubes to the mixed solvent caused selective etching from the center of the microtubes during solvent evaporation processes. This selective etching resulted in the formation of two half‐long microhorns from one microtube. The obtained fullerene microhorns exhibited preferential capture of silica particles over fullerene particles and polystyrene latex particles in the similar sizes. This selective capture capability of the fullerene microforms to hydrophilic particle could be used for sensing and the removal of problematic bio‐substances such as virus particles. Similarly, selective chemical etching of fullerene assemblies was very recently reported by site and face selective etching on 1D nanorods, 2D hexagon nanosheets, and 3D microcubes of fullerene molecules to give integrated hierarchical structures.^[^ ^67^ ^]^

**Figure 12:**
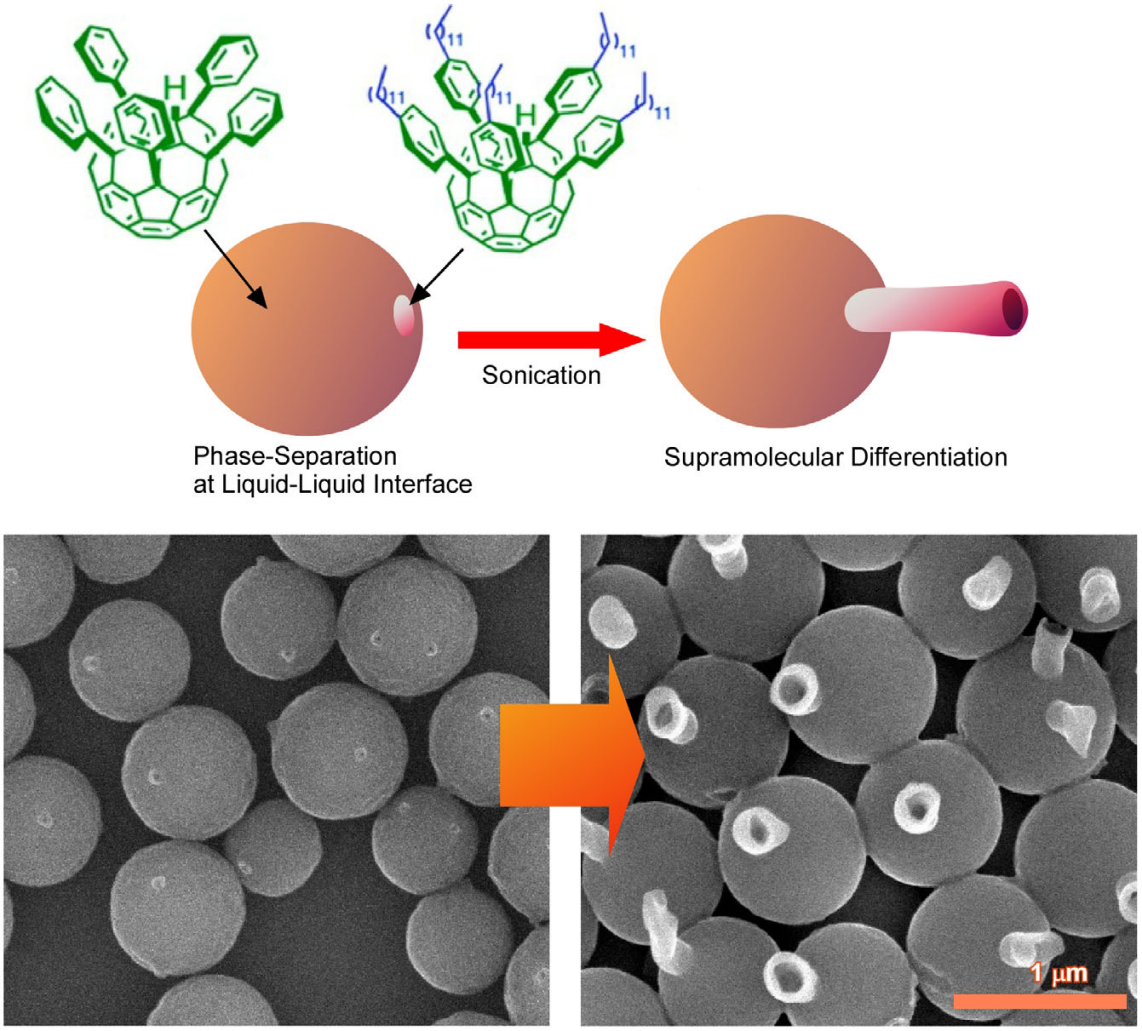
Bio‐like metamorphosis and differentiation from simple chemical objects, fullerene derivatives, pentakis(4‐dodecylphenyl)fullerene), and pentakis(phenyl)fullerene, at isopropyl alcohol/toluene interface, upon the addition of non‐equilibrium event of temporal irradiation of ultrasonic to self‐assembling process.

The addition of physical stimuli to self‐assembling processes can also lead to structural transformation to hierarchical structures. The following example demonstrated bio‐like metamorphosis and differentiation from simple chemical objects, fullerene derivatives, upon the addition of non‐equilibrium event of temporal irradiation of ultrasonic to self‐assembling process (**Figure** **12**).^[^ ^68^ ^]^ Co‐assembly of two kinds of fullerene derivatives, pentakis(4‐dodecylphenyl)fullerene) and pentakis(phenyl)fullerene, at isopropyl alcohol/toluene interface, first resulted in the formation of egg‐like spherical objects. Depending on the egg‐growth time, phase‐separated patch of pentakis(4‐dodecylphenyl)fullerene) was formed on the surface of pentakis(phenyl)fullerene. Temporal application of gentle sonication to shift equilibrium from a two‐phase liquid system to homogeneous solution induced the growth of a single tail of pentakis(4‐dodecylphenyl)fullerene) assembly from the single patch domain. Then, supramolecular metamorphic differentiation from egg‐like objects to tadpole‐like objects was completed. Because the number of phase‐separated patch structures was determined by the equilibrium growth time and one tail generated from one domain, the shapes of egg‐tail structures (the numbers of tails from one egg) can be solely determined by timing of the equilibrium shifts by sonication as the time‐programmed process. These processes do not include any bio‐components and biological processes, but living‐cell‐like metamorphic differentiation from egg to tadpole can be re‐generated purely by the physico‐chemical nanoarchitectonics process.

The above‐mentioned examples demonstrate the formation of various morphologies from 0D object fullerene molecules, which reveals high potential of nanoarchitectonics processes. Of course, nano‐units for structural construction are not limited to fullerene molecules. Various structural units, including inorganic nanomaterials, organic molecules, and biomolecules, are used for the architecting process from molecular level to materials size.^[^ ^69^ ^]^ Self‐assemblies based on specific interactions, such as hydrogen bonding in peptide assembly^[^ ^70^ ^]^ and DNA assembly^[^ ^71^ ^]^ and metal coordination in metal–organic frameworks (MOFs)^[^ ^72^ ^]^ and coordination polymers,^[^ ^73^ ^]^ have been extensively researched. These research activities can be merged into the nanoarchitectonics research as suggested from recent review articles.^[^ ^74^ ^]^

**Figure 13:**
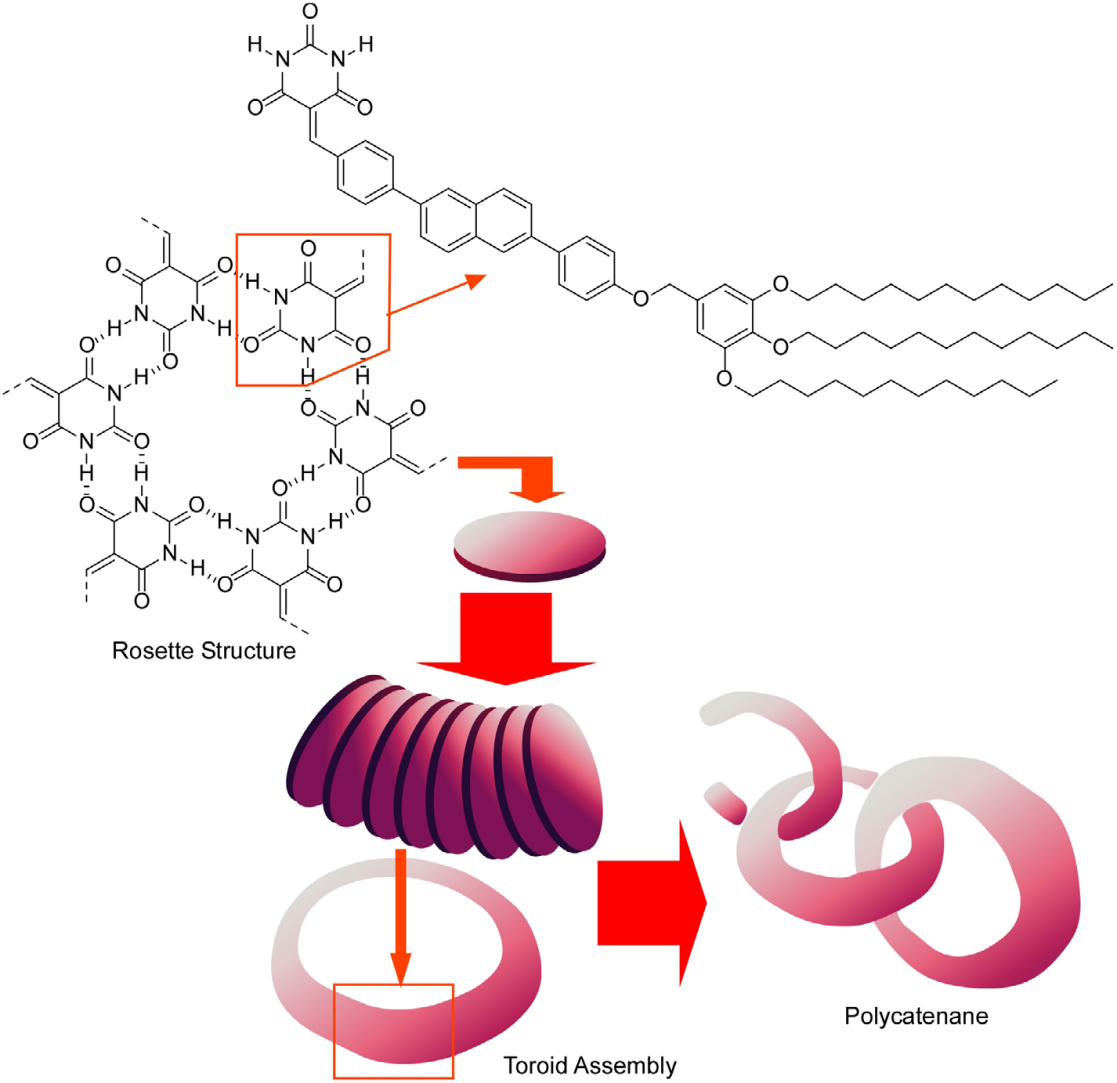
Significantly extended catenane linkages from hydrogen‐bond‐capable molecular units where supramolecular rosettes were assembled into supramolecular ring structures of toroids and were further entangled into catenane motif.

For example, beautiful examples on hierarchical self‐assembly from molecular units with self‐complementary hydrogen bonding capability were very recently reported by Yagai and co‐workers (**Figure** **13**).^[^ ^75^ ^]^ Including five‐toroid‐linked catenane as nanolympiadane, significantly extended catenane linkages from hydrogen‐bond‐capable molecular units were formed. Hierarchical assembly was started from the formation of unit rosette structure from barbituric acid derivative through complementary hydrogen bonding. The supramolecular rosettes were assembled into supramolecular ring structures of toroids that were further entangled into catenane motif. The catenane formation with high yields would be originated in nucleation of the second rosette at close vicinity to the preformed first catenane. Because extension of catenane structures is kinetically controllable depending on formation of supramolecular polymerization, non‐equilibrium factors such as sequences of injection of solution of nano‐units of barbituric acid derivative molecules have significant effects. Ten portion continuous injection of component solution with 1 s interval results in hugely extended polycatenation. Polycatenanes with 17, 18, and 22 linked toroids were actually observed. Some cooperative factors are surely working in the polycatenation process.

**Figure 14:**
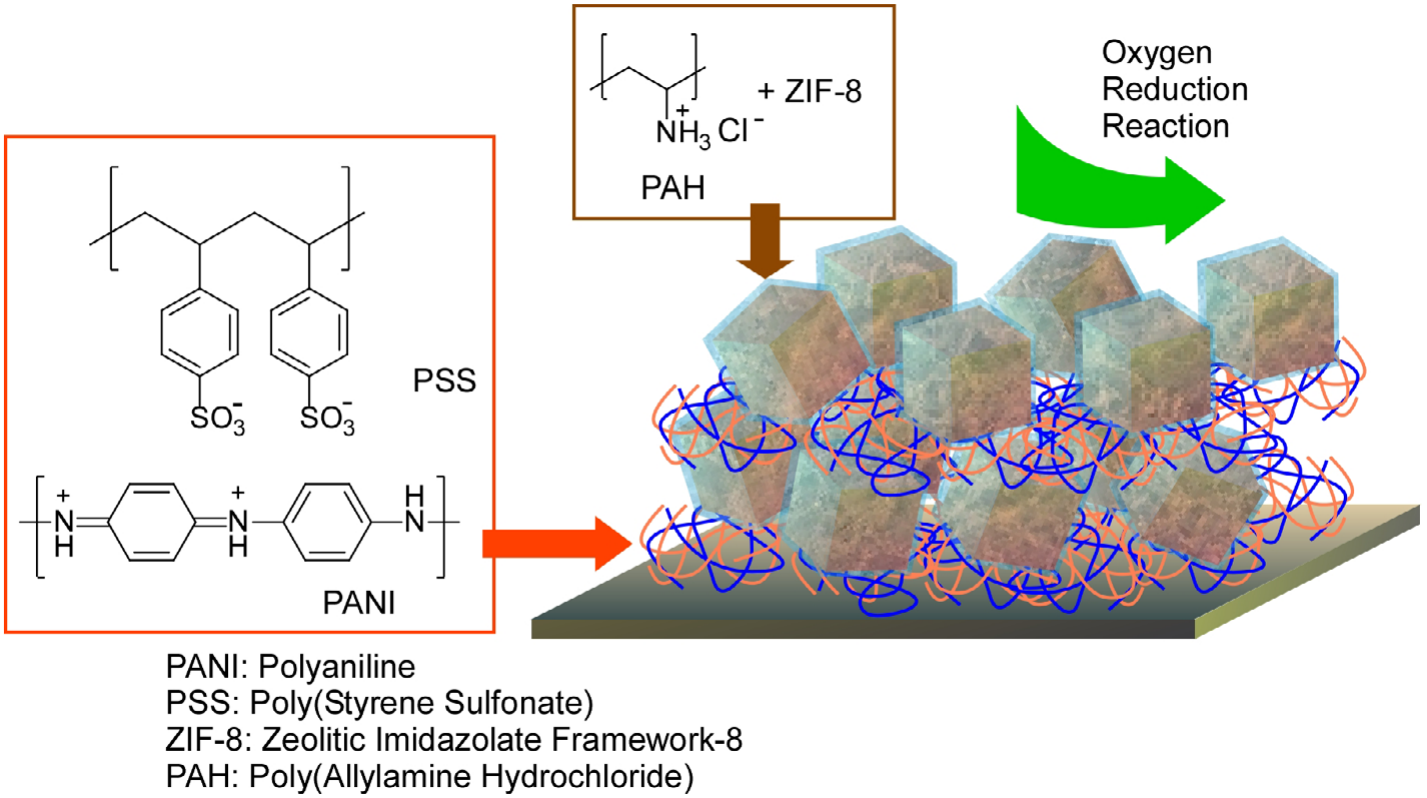
Hierarchical integration of conductive polymers with MOF structures using LbL assembly of two kinds of colloidal assemblies: anionic PANI/PSS colloidal particles and ZIF‐8 colloidal particles modified with cationic PAH.

Various functional material system with certain hierarchic structures have been recently reported with the concept of nanoarchitectonics. Coupling with non‐equilibrium fabrication methods such as the LB method and assembly is often advantageous to fabricate hierarchical functional materials. Azzaroni, Rafti, and co‐workers demonstrated the hierarchic integration of conductive polymers with MOF structures using LbL assembly as a nanoarchitectonics essence (**Figure** **14**).^[^ ^76^ ^]^ Fabrication of these hierarchical nanostructures started from synthesis of two kinds of colloidal assemblies: anionic polyaniline (PANI)/poly(styrene sulfonate) (PSS) colloidal particles and zeolitic imidazolate framework‐8 (ZIF‐8) colloidal particles modified with cationic poly(allylamine hydrochloride) (PAH). These electrically opposite colloidal substances were then assembled into nanoarchitected thin films by the LbL assembly. Enhanced electrochemical oxygen reduction reaction was observed for an electrode modified with the above‐mentioned hierarchical LbL structures in neutral pH aqueous environments. Synergic effect between oxygen‐adsorbing MOF (ZIF‐8) and electrocatalytic capability of the conductive polymer (PANI) was working within nanoscale vicinities. Enrichment of oxygen and electrochemical performance worked synergically in the fabricated hierarchic nanoarchitectures.

**Figure 15:**
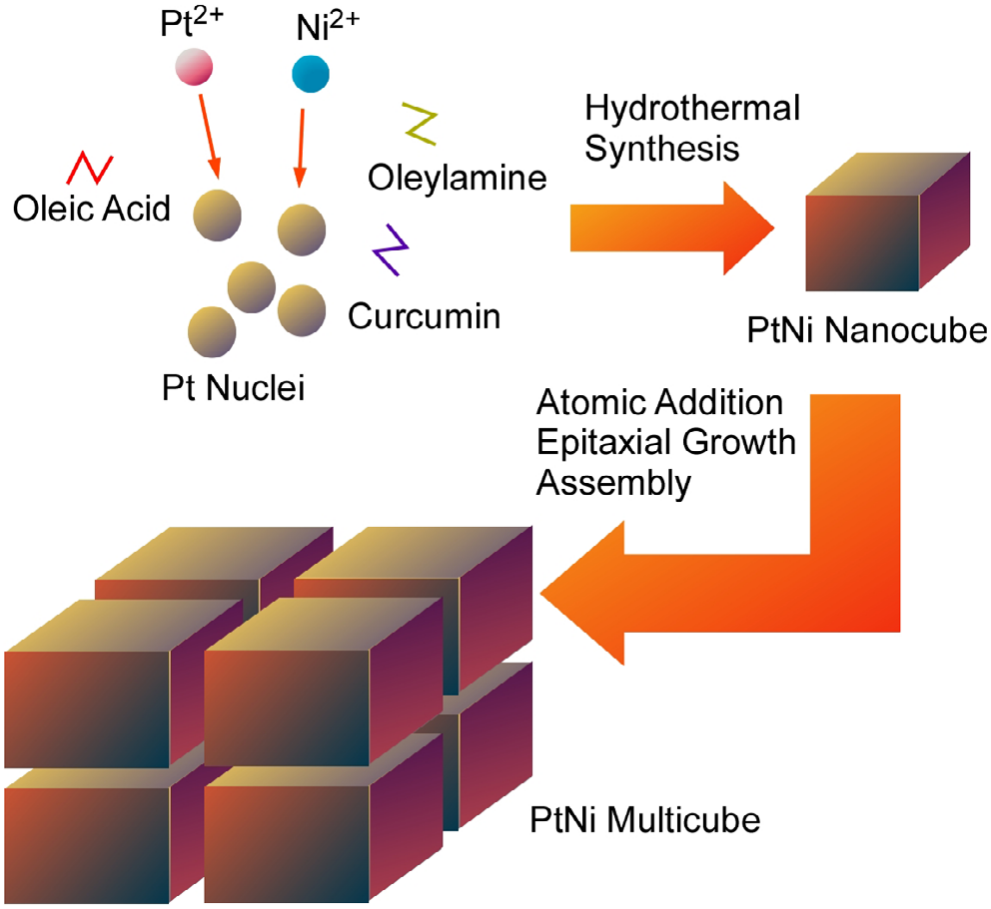
Multi‐dimensional nanoarchitectonics PtNi multicube structures through autocatalytic effect to form PtNi bimetallic nuclei, epitaxial growth, and assembly.

The formation of hierarchical nanoarchitectures is an important key for promoted reactions, which is often used in energy‐related applications. Elzatahry, Xu, and co‐workers proposed multi‐dimensional nanoarchitectonics PtNi multicube structures (**Figure** **15**).^[^ ^77^ ^]^ In the formation process, nucleation of Pt was initially promoted according to its standard reduction potential with the aid of ultrasonication. In hydrothermal treatments, spontaneous co‐reduction of Ni was induced through autocatalytic effect to form PtNi bimetallic nuclei. With the formed seeds, bimetallic PtNi cube structures were grown, and epitaxial growth with certain interactions resulted in hierarchic multicube architectures, because the formed hierarchic structures have various surface corners and inner cavities, leading to enhanced direct methanol oxidation reaction and ethanol oxidation reaction.

**Figure 16:**
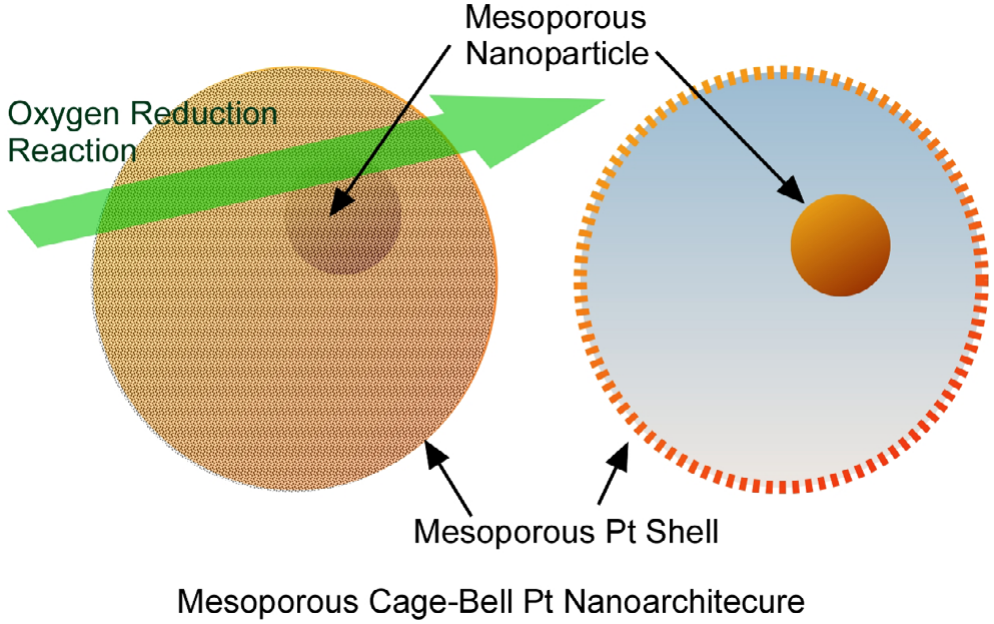
Cage‐bell nanoarchitectonics for efficient Pt catalysis in oxygen reduction reaction.

Pt‐based nanocatalysts have been paid much attentions with fuel‐cell‐related electrochemical applications, such as oxygen reduction reaction and methanol oxidation reaction. However, undesirable agglomeration of Pt nanostructures is problematic with actively loss and poor durability. Isolation in nanoarchitectures of active Pt catalysts would solve these problems. Xue, Wang, and co‐workers reported cage‐bell nanoarchitectonics for efficient catalysis in oxygen reduction reaction (**Figure** **16**).^[^ ^78^ ^]^ The cage‐bell nanoarchitectures were synthesized through step‐wise fabrications. Pt@SiO_2_ core–shell nanoparticles were first prepared and then covered with mesoporous Pt shell. Selective etching of SiO_2_ moiety resulted in hierarchical structure, mesoporous Pt nanocages with a Pt nanoparticle inside. The fabricated cage‐bell nanoarchitectures possess advantages of protection of Pt catalyst from agglomerations and maintenance of substrate diffusions, leading to superior catalytic activity and durability in oxygen reduction reaction.

**Figure 17:**
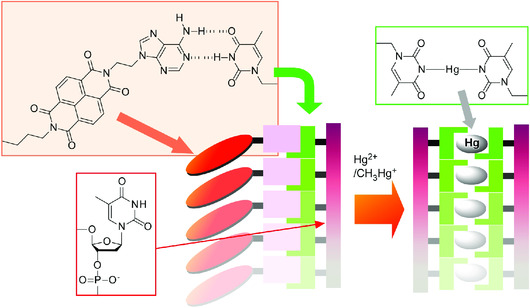
Dynamic changes of DNA‐based complex structures for sensitive mercury detections through quick transformation of the original co‐assemblies with organic semiconductor into a metallo‐DNA duplex with mercury.

Biomolecules have high capabilities in structure formation as seen in assemblies of amino acids, peptides, and nucleic acids. Biomolecular assemblies sometimes play important roles in nanoarchitectonics processes. Govindaraju and co‐workers demonstrated the dynamic changes of DNA‐based complex structures for sensitive mercury detections as a strategy for nanoarchitectonics of small molecule and DNA (**Figure** **17**).^[^ ^79^ ^]^ Chiral co‐assembly between the adenine‐conjugated organic semiconductors, adenine‐conjugated naphthalenediimide and deoxyribo‐oligothymidine, was used as a sensing complex with tunable chiroptical and electrical properties. The presence of mercury ions in water induced quick transformation of the original co‐assemblies into a metallo‐DNA duplex with mercury. This transformation was occurred accompanied with chiro‐optical and conductivity responses to enable subnanomolar‐level detection of mercury ions in water. DNA‐based nanoarchitectonics with hierarchical structures would be useful for novel optoelectronic devices and highly sensitive sensors.

### 3.3 Bridging Nano and Macro at Interfaces: Access to Life and Mimic of Life Architecture

**Figure 18:**
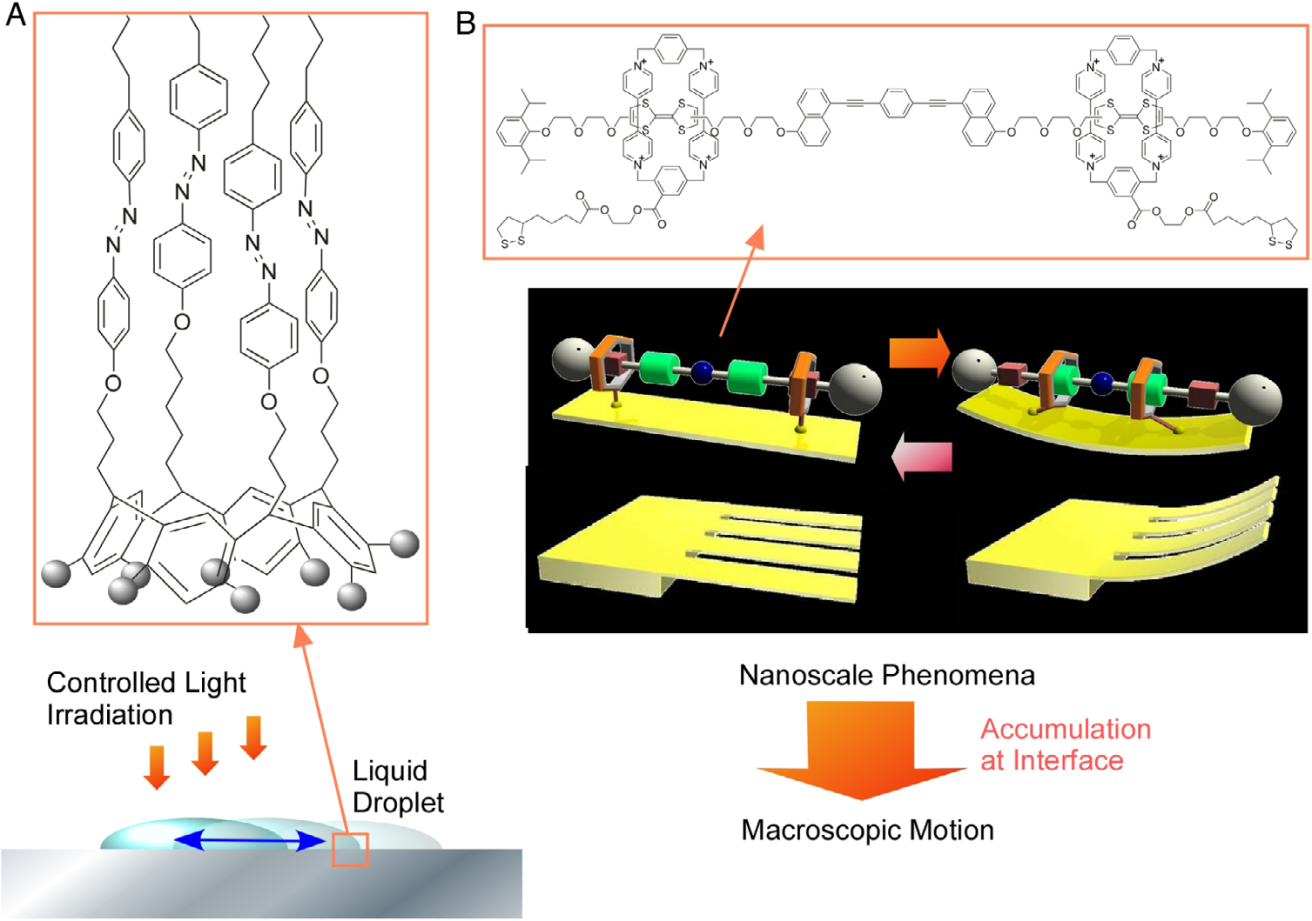
Accumulation of nano‐actions at interfaces as effective ways to induce macroscopic motions: A) motional control of liquid droplet by isomerization of monomolecular layer of photo‐active molecules and B) deformation of cantilever upon group actions of molecular machines.

One of the important aims of the nanoarchitectonics concept is establishment of all‐scale‐region understanding on creation of functional material systems. Although step‐wise and/or bottom‐up approaches from nanoscale units to macroscale visible‐size systems are principal strategies in nanoarchitectonics, methodology for direct bridging between nano and macro should also have important contributions. For the latter approach, interfacial environments have crucial roles as media to bridge nano and macro.^[^ ^80^ ^]^ Conversion of nanoscale actions to macroscopic functions needs huge accumulation of the former nano‐actions. Rather than random happening of nano‐actions in bulk media, accumulation of nano‐actions at interfaces is effective ways to induce macroscopic motions and actions from nano‐scale phenomena (**Figure** **18**).^[^ ^81^ ^]^ Deformation of cantilever upon group actions of molecular machines immobilized at the surface of cantilever was actually demonstrated^[^ ^82^ ^]^ as well as orientation control of liquid crystalline phase^[^ ^83^ ^]^ and motional control of liquid droplet^[^ ^84^ ^]^ by isomerization of monomolecular layer of photo‐active molecules at the interface.

**Figure 19:**
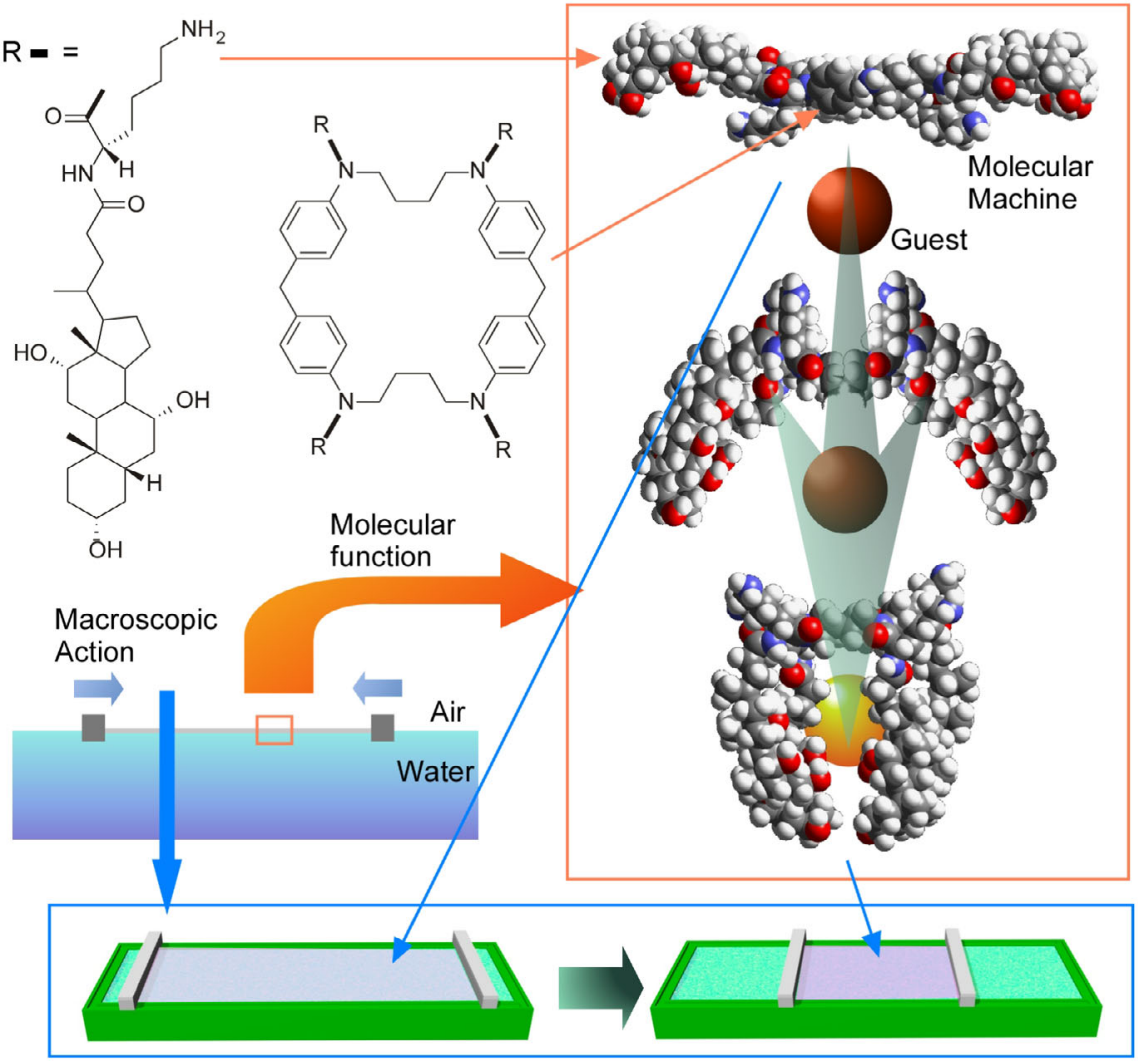
Mechanical compression and expansion of a Langmuir monolayer of molecular machines (steroid cyclophane molecules) embedded at the air–water interface for reversible capture and release of guest molecules from water subphase, respectively.

Interfacial media also have important roles in nano‐macro connection in opposite direction, i.e., control of nano‐functions upon macroscopic actions.^[^ ^85^ ^]^ For example, the controls of molecular machines by macroscopic mechanical actions were demonstrated at the air–water interface.^[^ ^86^ ^]^ Mechanical compression and expansion of a Langmuir monolayer of molecular machines (steroid cyclophane molecules) embedded at the air–water interface induced reversible capture and release of guest molecules from water subphase, respectively (**Figure** **19**).^[^ ^87^ ^]^ Dynamic interfaces, such as the air–water interface, are media to possess both macroscopic motional freedom in lateral direction and molecularly confined circumstance in thickness direction.^[^ ^88^ ^]^ Macroscopic motions and molecular actions can be coupled at dynamic interface. Therefore, control of molecular machines by hand‐motion‐like macroscopic actions^[^ ^89^ ^]^ becomes possible at dynamic interfacial media.

**Figure 20:**
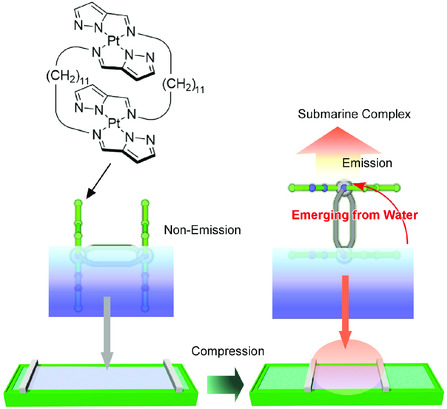
Submarine emission mechanism upon orientational changes of coordination complex molecules embedded on water as a Langmuir monolayer by external macroscopic forces.

In addition, interfacial environment modulates various physical and chemical properties of the embedded molecules. For example, molecular recognition capabilities at the air–water interface are much enhanced (10^6^ to 10^7^ times in some cases) as compared with those in bulk water.^[^ ^90^ ^]^ Huge difference of dielectric natures of two phases forming interfacial environment can be connected in drastic changes of optoelectrical properties. Coordination complex molecules embedded on water as a Langmuir monolayer change their fluorescence emission upon compression of the monolayer by external macroscopic forces. Subtle changes of molecular disposition at the air–water interface resulted in exposure of chromophore part to low‐dielectric phase accompanied with enhancement of fluorescence emission. This submarine emission mechanism^[^ ^91^ ^]^ is supported by nano–macro connections upon interfacial nanoarchitectonics (**Figure** **20**). Mechanical photo‐property controls of functional molecules embedded at dynamic interface were similarly reported as mechanical mechanically controlled indicator displacement assay.^[^ ^92^ ^]^ Deformation of indicator molecules upon mechanical compression at the air–water interface induced shifts in efficiencies of fluorescence resonance energy transfer between chromophores to give mechanically adjustable glucose detection.

**Figure 21:**
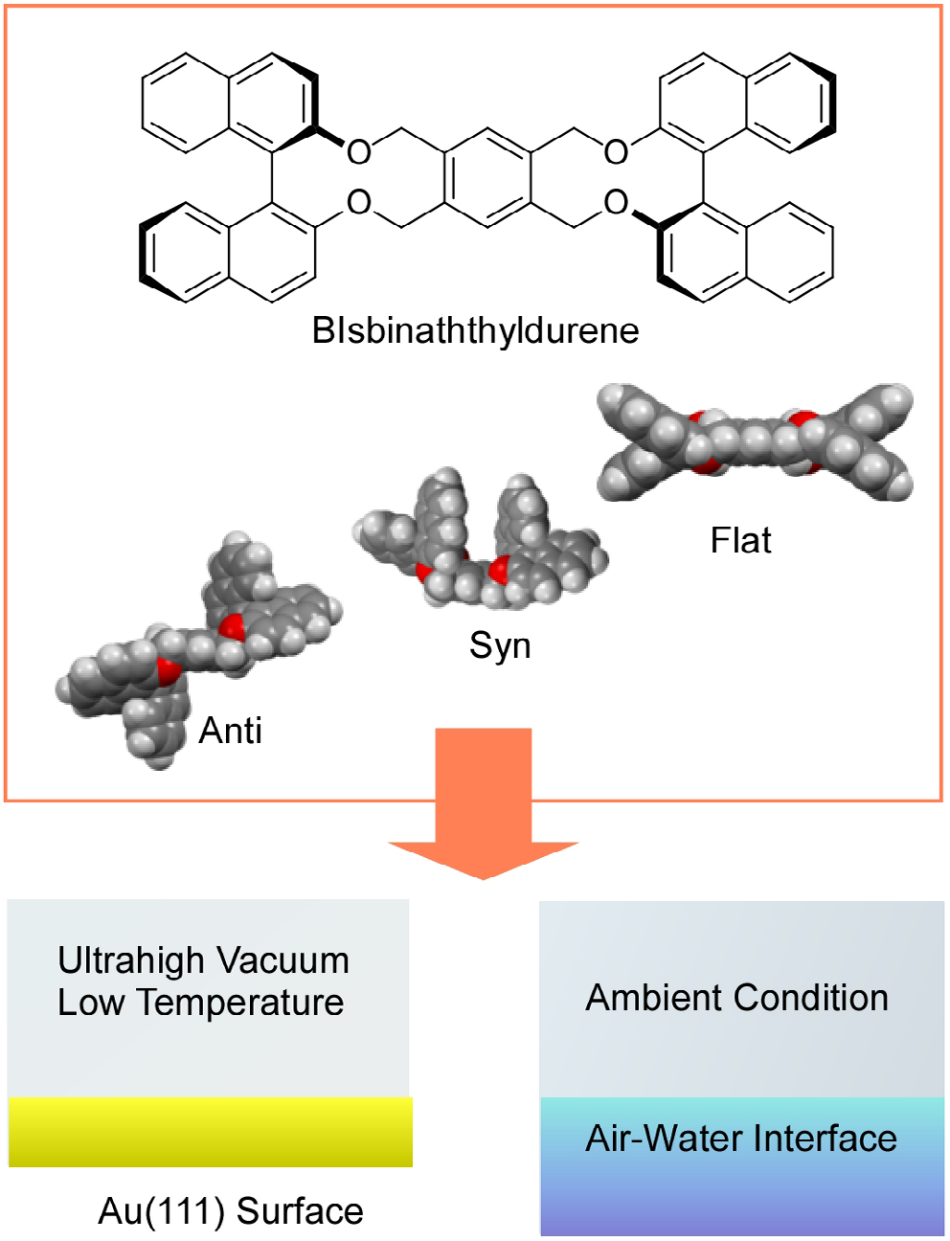
Investigations on conformational changes of a bisbinaphthyldurene molecule having two binaphthyl groups connected through a central durene moiety theoretically and experimentally at various environments including air–solid interface at low temperature under high vacuum and the air–water interface under ambient conditions.

Natures of interfaces, such as gas–solid interface and gas–liquid interface, have significant influence on behaviors of molecules embedded at their interface. As model cases, conformational changes of a bisbinaphthyldurene molecule having two binaphthyl groups connected through a central durene moiety were theoretically and experimentally examined at various environments, including homogeneous solution, air–solid interface at low temperature under high vacuum,^[^ ^93^ ^]^ and the air–water interface under ambient conditions (**Figure** **21**).^[^ ^94^ ^]^ This molecule was initially designed and synthesized as a nanocar (molecular car) for the first world‐wide nanocar race in 2017.^[^ ^95^ ^]^ Density functional theory (DFT) calculations revealed that the bisbinaphthyldurene molecule can adopt five major conformers, anti‐1, anti‐2, syn‐1, syn‐2, and flat. Different conformations (anti‐1 and syn‐1) are taken in solution phase. At low temperature under ultrahigh vacuum, the bisbinaphthyldurene molecule assembled as syn dimers on a Au(111) surface just after vacuum deposition. One molecule from the dimer can be forced to be in the flat conformer by mechanical actions by a tip of STM. Except the flat conformer, energy differences between the other conformers stay within only a couple of kcal mol^−1^ as estimated by DFT calculations. Their distributions are flexibly changeable at dynamic air–water interface dependent of co‐existing matrix lipids. The anti‐1 and syn‐1 were preferably formed in less miscible hard lipid matrices, whereas well miscible soft lipid matrices gave opportunity to form the anti‐2 and syn‐2 conformers. These results indicate that molecular conformations can be adjustable depending on interfacial nanoarchitectonics. Nanoarchitectonics design of interfacial environments would be an important key to control molecular machines that are highly expected to be used in membrane environments such as living cell surfaces.^[^ ^96^ ^]^

**Figure 22:**
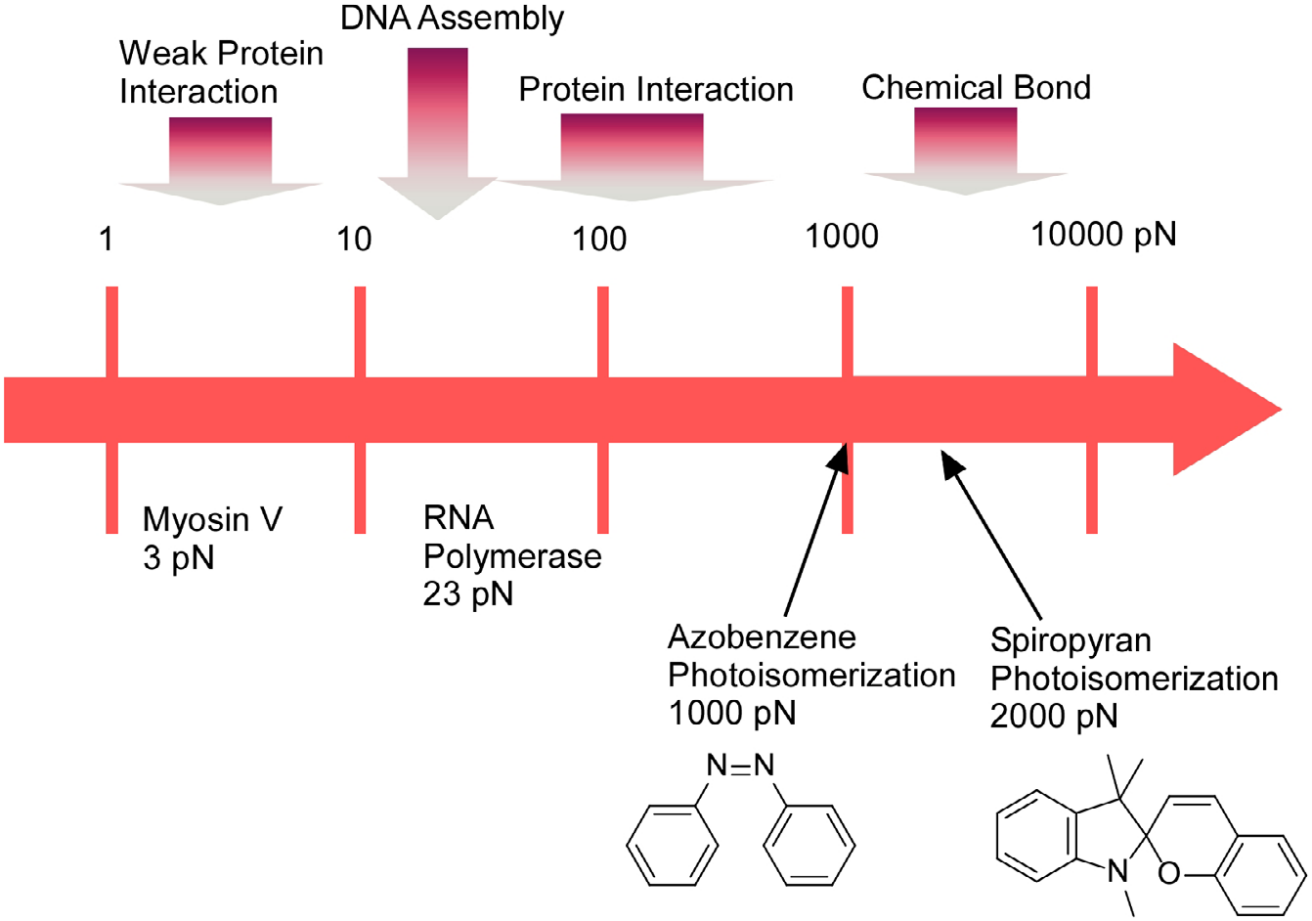
Required forces estimated for various molecular events.

Required forces for various molecular events are summarized in **Figure** **22**.^[^ ^97^ ^]^ This diagram roughly showed clear tendency. Biological mechanisms often utilize small force and small energy processes as compared with molecular transformation of artificial systems such as photo‐isomerization. Many molecular mechanisms are working with small force and tiny energy consumption under influences of thermal fluctuation. Bio‐functions based on delicate conformational changes are much different from artificial molecular machines working with distinct structural changes upon photo‐isomerization and strong interactions. Controls of molecular conformation through mechanical processes at dynamic interface are close to biological delicate processes rather than most of artificial supramolecular systems. In fact, investigations with model molecular machines, amphiphilic binaphthyl molecule as molecular pliers, at the air–water interface revealed that required energy for pliers operation and supplied energy by compression are very small (1 kcal mol^−1^ level).^[^ ^98^ ^]^ Separate estimation on mechanical processes at the air–water interface suggested 0.5 to 35 pN level force per molecules. Molecular manipulations at the air–water interface are very close to biological delicate systems and are much different from most of artificial molecular systems such as photo‐responding systems.

**Figure 23:**
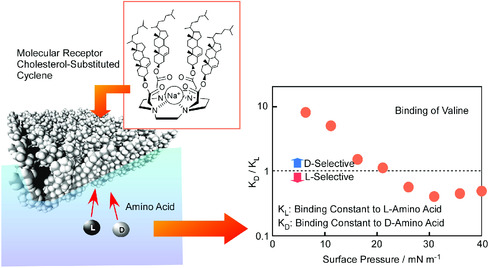
Application of macroscopic mechanical forces laterally to a Langmuir monolayer of *N*‐substituted cyclen, containing a 1,4,7,10‐tetraazacyclododecane core with four cholesteric side arms as molecular receptor, for pressure‐dependent chiral recognition of amino acids in aqueous subphase.

As demonstrations of delicate controls of molecular systems at dynamic interface, mechanical tuning of molecular receptors was investigated at the air–water interface.^[^ ^99^ ^]^ Application of macroscopic mechanical forces laterally to a Langmuir monolayer of *N*‐substituted cyclen, containing a 1,4,7,10‐tetraazacyclododecane core with four cholesteric side arms as molecular receptor, resulted in pressure‐dependent chiral recognition of amino acids in aqueous subphase (**Figure** **23**).^[^ ^100^ ^]^ Delicate conformational changes of the molecular receptor switched binding preference to L‐ or D‐amino acid. Interfacial mechanical tuning of another molecular receptor, cholesterol‐substituted triazacyclononane, achieved efficient selection between uracil derivative from thymine derivative,^[^ ^101^ ^]^ although they have single methyl‐group difference and cannot be distinguished naturally occurring DNA and RNA. Delicate molecular tuning makes delicate molecular discrimination possible.

**Figure 24:**
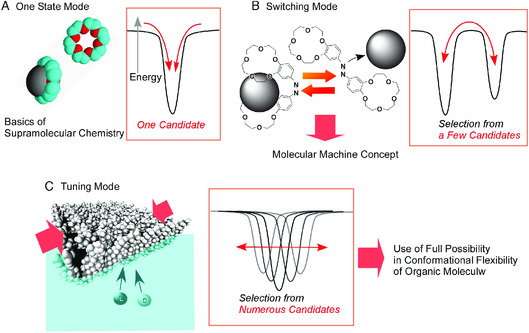
Three major modes of molecular recognition: A) one state mode, B) switching more, and C) tuning mode.

These approaches on molecular recognition are based on capability of conformational changes of flexible organic receptor molecules, although precise structural motifs of host–guest complex upon crystallographic analyses are often discussed in mainstream of supramolecular chemistry. Three modes of molecular recognition are summarized in **Figure** **24**.^[^ ^102^ ^]^ The most basic molecular recognition mode utilizes one most stable structure between host (receptor) and guest. This is a starting point of supramolecular chemistry and was awarded with the Nobel prize in 1987.^[^ ^103^ ^]^ Concept on switching of receptor structure by external stimuli was initiated by Shinkai et al. using photo‐isomerization of receptors.^[^ ^104^ ^]^ This is the second mode of molecular recognition that can be switchable between two or more stable states. This molecular switching concept also leads to molecular machines, which was awarded with the Nobel prize in 2016.^[^ ^105^ ^]^ Most of molecular machine operations are based on switching between a few stable state upon inputs of external stimuli. Unlike these previous examples, delicate molecular tuning of receptor at dynamic interface is based on continuous changes of receptor conformations. Among numerous conformational candidates, the most desirable receptor conformation is selected. This mode of molecular recognition is assigned as the third mode of molecular recognition. It would have similar nature of biological molecular mechanisms.

**Figure 25:**
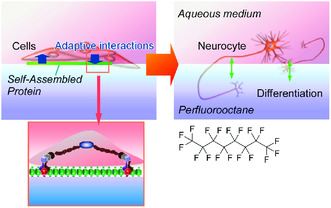
Interfacial culture system to human mesenchymal stem cells using perfluorooctane as organic phase where neural differentiation was promoted upon delicate interaction with the formed protein assembly.

Delicate molecular tuning at nanoarchitected organization at the air–water interface has common features with biological systems. This interfacial nanoarchitectonics strategy can be directly applied to regulation of living cells. Recently, cultures of living cells and their differentiation controls at interfaces between aqueous cell culture medium and fluorocarbon fluid have been demonstrated by Minami et al.^[^ ^106^ ^]^ Jia et al. further applied this interfacial culture system to human mesenchymal stem cells using perfluorooctane as organic phase (**Figure** **25**).^[^ ^107^ ^]^ Because of the presence of additional fibronectin, delicate protein monolayer was spontaneously formed at the water–fluorocarbon interface. The formed protein monolayer and human mesenchymal stem cells have mutual interaction through mechanical communications. Flexible nature of liquid–liquid interface provides opportunity of free mutual interaction in both direction from cell to protein assembly and from protein assembly to cell. Neurodifferentiation was promoted for human mesenchymal stem cells accompanied with morphological change of the flat protein film to hierarchical structure containing small fibers. This delicate interactive system does not require expensive liquid factors, such as cytokines, for induction of cell differentiation. The demonstrated system has high potentials in application to inexpensive processes for cell engineering.

**Figure 26:**
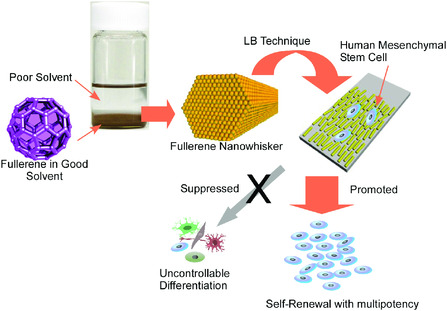
Culture system for human mesenchymal stem cells both capable of enhanced self‐renewal properties and retained multipotency using a solid interface covered with aligned fullerene nanowhiskers by the LB method.

Of course, cell culture and organization at solid interface have great potential for medical usages as seen excellent cell‐sheet engineering led by Kobayashi and Okano.^[^ ^108^ ^]^ Solid surface with nanoarchitected structures was also subjected to cell regulation. For example, aligned 1D fullerene nanowhiskers transferred onto a solid surface by the conventional LB methods^[^ ^109^ ^]^ or vortex LB method^[^ ^110^ ^]^ exhibited capability of cell alignments and differentiation control. Very recently, Song et al. successfully demonstrated the culture system for human mesenchymal stem cells both capable of enhanced self‐renewal properties and retained multipotency using a solid interface covered with aligned fullerene nanowhiskers by the LB method (**Figure** **26**).^[^ ^111^ ^]^ Well optimized contact of human mesenchymal stem cells on hydrophobic surface of nanoarchitectured nanocarbon arrays resulted in certain extent of suppression of focal adhesions with small cytoskeletal tension, which is advantageous for long‐term retention of multipotency and expansion capability for human mesenchymal stem cells.

**Figure 27:**
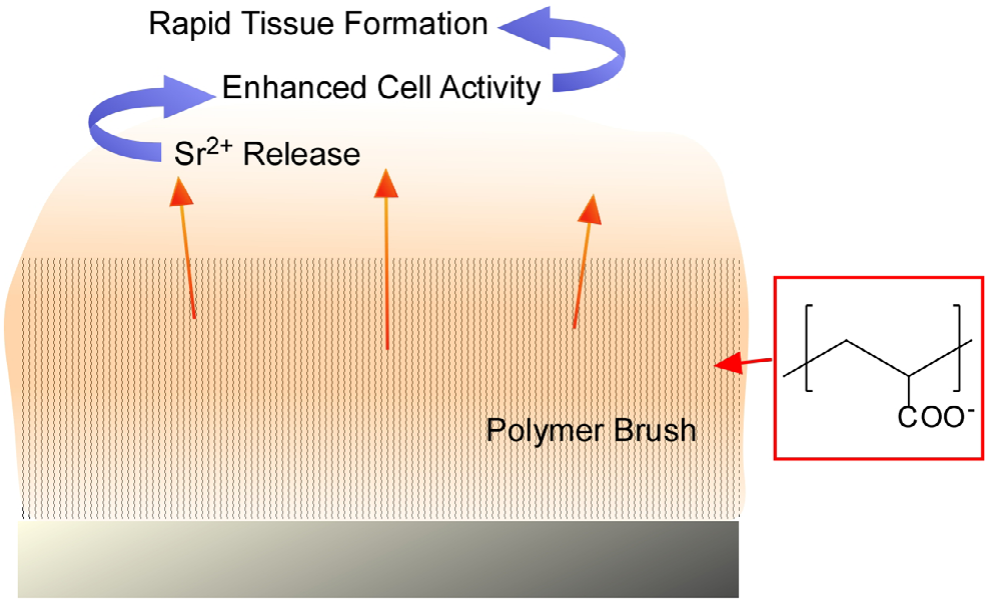
Soft nanoarchitectonics approach using polymer brush grown from TiO_2_ surface for promoted osseointegration upon slow delivery of Sr^2+^ ions.

Gregurec, Moya, and co‐workers demonstrated the soft nanoarchitectonics approach using polymer brush grown from TiO_2_ surface for promoted osseointegration (**Figure** **27**).^[^ ^112^ ^]^ The polymer brush was made of polyacrylic acid that can confine Sr^2+^ ions and make slow delivery of Sr^2+^ ions that are essential for bone remodeling. As compared with base TiO_2_ surface, enhanced initial adhesion of osteoblast cells and rapid cell tissue formation were confirmed on soft‐nanoarchitected surface with polyacrylic acid brushes with higher Sr^2+^ ion content.

**Figure 28:**
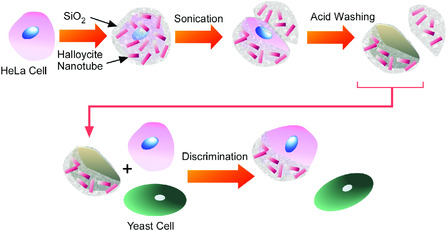
Fabrication of cell‐recognizing materials using HeLa cells as sacrificial template using silica/halloysite composites.

Cell surfaces are used for media for interfacial nanoarchitectonics as cell surface engineering nanoarchitectonics. Fakhrullin and co‐workers fabricated cell‐recognizing materials using HeLa cells as sacrificial template (**Figure** **28**).^[^ ^113^ ^]^ With the aid of a polyelectrolyte nanolayer and silica/halloysite composites, these were prepared on the HeLa cells. Disintegration with sonication and dissolution of cell debris upon washing with acid resulted in inorganic materials with imprinted structure of cell surface information. Using the imprint‐nanoarchitected materials, discrimination between HeLa cells in cell growth media supplemented with yeast cells was examined. Sophistication of this strategy is potentially useful for discrimination between cancer cells and normal cells.

**Figure 29:**
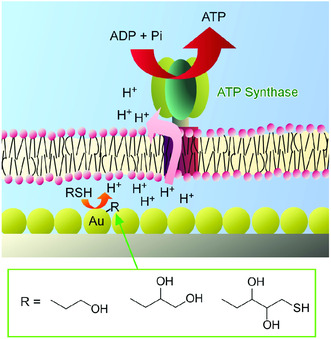
Layered hybrid systems with lipid bilayer membrane including motor protein adenosine triphosphate (ATP) synthase and thin film of gold nanoparticles on a glass substrate for conversion from adenosine diphosphate (ADP) to ATP upon formation of SAM on the surface of the gold nanoparticles accompanied with proton generation.

Essence of interfacial nanoarchitectonics can be actually recognized in biological systems as seen in highly integral organization of photosynthetic systems at cell membranes. Fabrications of nanoarchitected organization including functional biomolecules at interfacial environments would be effective ways to produce functional systems bio‐comparable high functional systems. Recently, Li et al. successfully fabricated layered hybrid systems with lipid bilayer membrane including motor protein ATP synthase and thin film of gold nanoparticles on a glass substrate (**Figure** **29**).^[^ ^114^ ^]^ In this system, alkane thiol molecules were used as chemical fuels for conversion from ADP to ATP. Added alkane thiol molecules formed SAM on the surface of the gold nanoparticles accompanied with proton generation. The latter process induced the formation of proton gradient across the lipid bilayer that motivated the function of the motor protein ATP synthase to convert ADP to ATP. Layered nanoarchitectonics design with appropriate sequence can result in relayed chemical conversion to provide important ATP molecules from input of conventional thiol molecules. More sophisticated nanoarchitectonics design to couple functional biological molecules with artificial nanoarchitectures would have further possibilities to create biomimetic energy and materials conversion systems.

Fabrication of bio‐like high functional systems through organization of nano‐units is one of the important targets in nanoarchitectonics. Coupling of nano/micro‐actions and integration of various functional units at interfacial environments are important keys to accomplish this aim.

## 4 Perspectives

In this short review article, essences of nanoarchitectonics approach are explained with several new examples. The nanoarchitectonics is a concept that comes next to nanotechnology. Based on facts and knowledge in nanoscale objects explored by nanotechnology, functional material systems are constructed using nano‐units with the aid of the other research fields, such as organic chemistry, supramolecular chemistry, materials science, and biology. Introduction of nanotechnological essences to material construction can produce unusual functional systems, such as brain‐like information processing based on atomic‐level reactions, diffusions, and aggregations. Probe‐tip‐mediated processes can be used for organic reactions with precise site selectivity. Coupling of equilibrium self‐assemblies and non‐equilibrium fabrication processes resulted in variously structured and hierarchical functional structures even from simple 0D nano‐units such as fullerenes. Especially, interfacial nanoarchitectonics directly bridges nanoscopic functions and macroscopic actions including facile contact with nanostructures and living cells. Materials nanoarchitectonics processes at interfacial media are also beneficial to couples functional biounits and artificial nanostructures. Nanoarchitectonics would work in all the scale lengths to construct functional materials systems.

**Figure 30:**
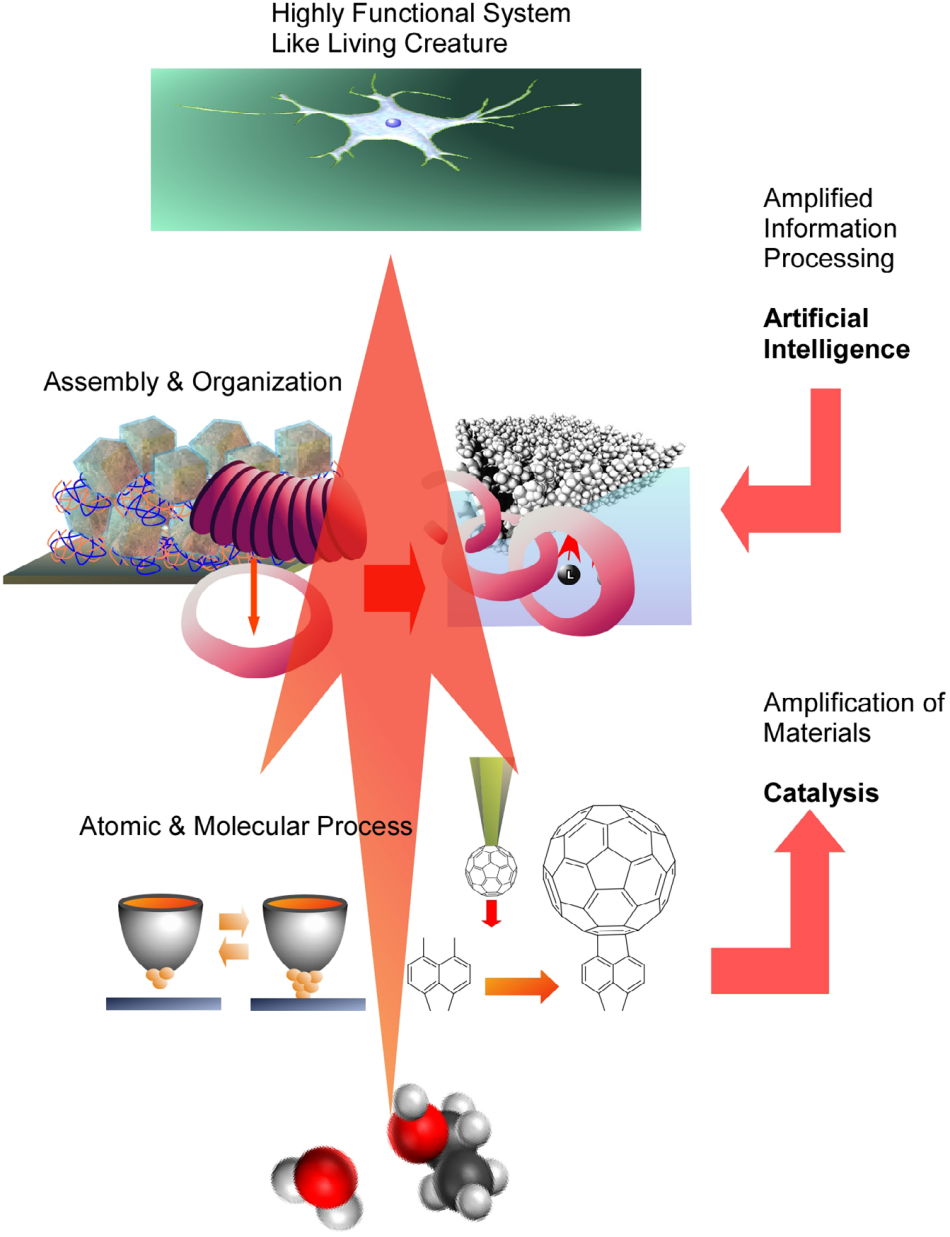
Summary and possible future directions of nanoarchitectonics.

In **Figure** **30**, the above‐mentioned features together with possible future directions of nanoarchitectonics are summarized. Establishment of general methodology to totally cover materials fabrication from nano to macro is, in fact, tough target. For near future goal of nanoarchitectonics, some mechanisms such as effect amplification would be necessary to reflect nanoscale phenomena to bulk‐size outputs. Catalysts would be desirable targets for near‐future goal of the nanoarchitectonics approach.^[^ ^115^ ^]^ As well known, catalysts, including conventional catalysts,^[^ ^116^ ^]^ enzymes,^[^ ^117^ ^]^ and artificial enzymes,^[^ ^118^ ^]^ have intrinsic capabilities of amplified materials conversions. Recent research progresses have revealed high potentials of nanostructured catalysts including single atom catalysts.^[^ ^119^ ^]^ Therefore, regulations of atomic arrangements including rational organization of multiple catalytic sites possible lead to sophisticated conversions of materials and energies in long sequential and cascade modes with outputs of reasonable quantities. Not limited to conventional catalysis, the similar strategies can be applied to electrochemical catalysts to achieve highly efficient energy production, conversion, and management.

Nanoarchitectonics capabilities to assemble and harmonize various functions and effects can be more refined by the aid of emerging technology related to artificial intelligence. Optimum selection of component nano‐units and applied processes for target functional materials from numerous experimental facts and theoretical estimations would be rationally done with the computer‐based technology. Approaches based on machine learning were already applied to materials science and related fields.^[^ ^120^ ^]^ The working principle of machine learning to find best choice from numerous possibilities would be much closer to approaches for materials nanoarchitectonics with combined processes rather than conventional material production. It may also be compared with long‐term evolution processes in nature where optimum functional systems (living creatures) are created through optimization and organization from simple molecular initiators. Combination of nanoarchitectonics concept and artificial intelligent technologies can accomplish preparation of highly functional materials within very short time instead of over billions of years.

If we assume that the most advanced materials systems are living creatures, the final goal of nanoarchitectonics approach would be the creation of highly functional materials similar to living creatures. Revolution from nanotechnology to nanoarchitectonics would enable us to develop methodology for material production comparable to evolution process in nature. The establishment of processes equivalent to billions‐years of natural evolution within a few decades would be the final goal of nanoarchitectonics.

## Conflict of Interest

The author declares no conflict of interest.
